# The Plasma Membrane Ca^2+^ Pump PMCA4b Regulates Melanoma Cell Migration through Remodeling of the Actin Cytoskeleton

**DOI:** 10.3390/cancers13061354

**Published:** 2021-03-17

**Authors:** Randa Naffa, Rita Padányi, Attila Ignácz, Zoltán Hegyi, Bálint Jezsó, Sarolta Tóth, Karolina Varga, László Homolya, Luca Hegedűs, Katalin Schlett, Agnes Enyedi

**Affiliations:** 1 Department of Transfusiology, Semmelweis University, H-1089 Budapest, Hungary; naffaranda@student.elte.hu (R.N.); toth.sarolta@med.semmelweis-univ.hu (S.T.); 2 Department of Biophysics and Radiation Biology, Semmelweis University, H-1094 Budapest, Hungary; padanyi.rita@med.semmelweis-univ.hu; 3 Department of Physiology and Neurobiology, Eötvös Loránd University, H-1117 Budapest, Hungary; ignacz.attila@ttk.elte.hu (A.I.); schlett.katalin@ttk.elte.hu (K.S.); 4 Institute of Enzymology, Research Centre for Natural Sciences, Magyar Tudosok krt.2, H-1117 Budapest, Hungary; hegyi.zoltan@bio-science.hu (Z.H.); jezso.balint@ttk.elte.hu (B.J.); homolya.laszlo@ttk.hu (L.H.); 5 Department of Anatomy, Cell and Developmental Biology, Eötvös Loránd University, H-1117 Budapest, Hungary; 6 Versys Clinics, H-1138 Budapest, Hungary; research@versysclinics.com; 7 Department of Thoracic Surgery, Ruhrlandklinik, University Clinic Essen, 45239 Essen, Germany; luca.hegedues@rlk.uk-essen.de

**Keywords:** plasma membrane Ca^2+^ ATPase 4b, BRAF mutant melanoma, actin cytoskeleton, cofilin, cell motility, metastasis

## Abstract

**Simple Summary:**

Earlier we demonstrated that the plasma membrane Ca^2+^ pump PMCA4b inhibits migration and metastatic activity of BRAF mutant melanoma cells, however, the exact mechanism has not been fully understood. Here we demonstrate that PMCA4b acted through actin cytoskeleton remodeling in generating a low migratory melanoma cell phenotype resulting in increased cell–cell connections, lamellipodia and stress fiber formation. Both proper trafficking and calcium transporting activity of the pump were essential to complete these tasks indicating that controlling Ca^2+^ concentration levels at specific plasma membrane locations such as the cell front played a role. Our findings suggest that PMCA4b downregulation is likely one of the mechanisms that leads to the perturbed cancer cell cytoskeleton organization resulting in enhanced melanoma cell migration and metastasis.

**Abstract:**

We demonstrated that the plasma membrane Ca^2+^ ATPase PMCA4b inhibits migration and metastatic activity of BRAF mutant melanoma cells. Actin dynamics are essential for cells to move, invade and metastasize, therefore, we hypothesized that PMCA4b affected cell migration through remodeling of the actin cytoskeleton. We found that expression of PMCA4b in A375 BRAF mutant melanoma cells induced a profound change in cell shape, cell culture morphology, and displayed a polarized migratory character. Along with these changes the cells became more rounded with increased cell–cell connections, lamellipodia and stress fiber formation. Silencing PMCA4b in MCF-7 breast cancer cells had a similar effect, resulting in a dramatic loss of stress fibers. In addition, the PMCA4b expressing A375 cells maintained front-to-rear Ca^2+^ concentration gradient with the actin severing protein cofilin localizing to the lamellipodia, and preserved the integrity of the actin cytoskeleton from a destructive Ca^2+^ overload. We showed that both PMCA4b activity and trafficking were essential for the observed morphology and motility changes. In conclusion, our data suggest that PMCA4b plays a critical role in adopting front-to-rear polarity in a normally spindle-shaped cell type through F-actin rearrangement resulting in a less aggressive melanoma cell phenotype.

## 1. Introduction

Melanoma is a form of skin cancer that originates from the neural crest derived melanocytes that produce melanin. It is a highly invasive type of cancer that tends to metastasize and causes death. Metastasis is a multistep process that requires migration of cells from the primary tumor to other sites in the body [1]. It has been widely acknowledged that changes in the actin cytoskeleton arrangement are essential for cells to migrate and metastasize [2]. Many proteins are involved in the regulation of actin dynamics and any alteration in their expression, activity, or localization may contribute to cellular transformation and tumorigenesis [3,4].

Several studies suggested that cytoplasmic free Ca^2+^ concentration plays a role in actin-based changes of cell polarity, chemotaxis, and motility [5,6,7]. In addition, Ca^2+^ is involved in actin rearrangement, focal adhesion turnover, invadopodia, and lamellipodia formation during cell migration [8,9]. Cells maintain Ca^2+^ homeostasis using a “molecular toolkit” that includes Ca^2+^ channels to allow Ca^2+^ to enter the cell and pumps or exchangers to remove excess Ca^2+^ for proper cell functioning. Changes in the expression of any of these tools can result in altered Ca^2+^ homeostasis leading to uncontrolled cell proliferation and metastasis [10].

Overexpression of Ca^2+^ channels has been implicated in the progression of several cancer types [11]. A recent study [12] reported elevation of T-type calcium channels (TTCCs) Cav3.1 in BRAFV600E mutant melanomas that promoted melanoma cell proliferation and migration. In bladder cancer, an increased level of transient receptor potential melastatin 8 (TRPM8) promoted cell proliferation, motility, and migration [13]. In metastatic prostate cancer, increased expression of the transient receptor potential cation channel subfamily V member 2 (TRPV2) resulted in enhanced cell motility through the invasion associated enzymes MMP9 and cathepsin B [14].

Cell motility and migration require filamentous actin (F-actin) rearrangements, and free Ca^2+^ concentration was found to play a role in this process [5,15]. The involvement of the transient receptor potential melastatin 4 (TRPM4) has been demonstrated in Ca^2+^ dependent actin cytoskeleton reorganization and migration of bone marrow-derived mast cells (BMMCs) [16]. Another study showed that transient receptor potential cation channel subfamily V member 4 (TRPV4) increased the migration of breast cancer cells via remodeling of the actin cytoskeleton through the Ca^2+^-dependent activation of AKT [17].

Several actin binding-proteins are affected by changes in cytoplasmic Ca^2+^ concentration [18]. An important regulator of actin dynamics is the actin severing protein cofilin [19]. Inhibition of cofilin activity may disrupt cell polarity, protrusion formation, and chemotaxis. Cofilin is a 19 kDa protein that catalyzes actin depolymerization but also acts as a polymerization factor through producing monomer actin for the generation of locomotory protrusions at the leading edge of migrating cells. Cofilin activity is regulated by phosphorylation at Ser3, and by binding to PIP_2_ or cortactin [20]. A study has reported that high concentration of free intracellular Ca^2+^ can induce cofilin dephosphorylation and activation through the calcium-dependent phosphatase calcineurin [21].

The plasma membrane Ca^2+^-ATPase (PMCA) is a key regulator of cytosolic Ca^2+^ concentration that expels Ca^2+^ from the cell using the energy of ATP. Four different genes (*ATP2B1-4*) encode PMCAs in mammals (PMCA1-4) from which more than twenty variants are transcribed as a result of alternative mRNA splicing [22]. PMCAs have been implicated in a variety of cancer types. While PMCA2 (*ATP2B2*) was upregulated in HER+ breast cancer tumors [23], PMCA4 (*ATP2B4*) was downregulated in colon and prostate cancers, and lymph node metastases in contrast to the relatively high PMCA4 protein level in normal tissues [24,25].

Our laboratory identified the PMCA4b variant as a putative metastasis suppressor using BRAF mutant melanoma cell models [26]. We found that in selected melanoma cells with RAS or BRAF mutations PMCA4b was expressed at low levels. Inhibition of BRAF, MEK, or p38 MAPK, however, increased the expression of PMCA4b without affecting the expression of other Ca^2+^ pumps or Ca^2+^ channels [26,27]. The enhanced PMCA4b expression caused a dramatic change in cell motility without having a significant effect on proliferation, characteristic features of metastasis suppressors. We also identified additional PMCA4b expression inducers, such as the histone deacetylase inhibitors some of which are already in clinical use in a variety of melanoma, breast, and colon cancer cells [28,29,30].

Since the free Ca^2+^ ion level is an important modulator of actin cytoskeletal dynamics, the goal of the present study was to investigate whether PMCA4b, by controlling intracellular Ca^2+^ levels, acts through the actin cytoskeleton in reducing motility of BRAF mutant melanoma cells. We investigated the role of PMCA4b activity and trafficking in maintaining the integrity of the actin cytoskeleton and we studied the formation of cell–cell connections, lamellipodia and stress fibers in cells expressing wild type and mutant PMCA4b proteins. We show that polarized distribution of PMCA4b maintains a gradient of cytosolic free Ca^2+^ levels and induces cofilin redistribution to the leading edge that ultimately leads to a low motility melanoma cell phenotype.

## 2. Materials and Methods

### 2.1. Cell Culture

BRAF (V600E) mutant melanoma (A375) and breast cancer (MCF-7) cells were purchased from ATCC. Cells were cultured in Dulbecco’s modified Eagle’s medium (DMEM) (Lonza, Walkersville, MD, USA) supplemented with 10% fetal bovine serum (FBS) (Thermo Fisher Scientific, Waltham, MA, USA), 1% penicillin–streptomycin (Lonza), and 2 mM L-glutamine (Lonza) in humidified 5% CO_2_ incubator at 37 °C.

### 2.2. Chemicals and Reagents

Cytochalasin D (cytD), calcium ionophore (A23187), and Phalloidin-TRITC were dissolved in DMSO and stored at −20 °C as a stock solution. The final DMSO concentration in the experiment did not exceed 0.01%. All reagents were purchased from Sigma-Aldrich (St. Louis, MO, USA).

### 2.3. DNA Constructs

The DNA plasmid of pmCherry-C1 and pEGFP-actin was purchased from Clontech Laboratories Inc., Mountain View, CA, USA. The mCherry-PMCA4b plasmid was generated as described previously [31]. The trafficking mutant pEGFP-PMCA4b-L^1167-1169^A construct was prepared previously [31]. The SB-CAG-GFP-PMCA4b-CAG-Puromycin and SB-CAG-GFP-PMCA4b-LA-Puromycin constructs were generated, as described [26]. The non-functional mutant mCherry-PMCA4b-DE was created by introducing the D672E point mutation into the mCherry-PMCA4b and GFP-PMCA4b plasmids using QuikChange II Site-Directed Mutagenesis Kit (Stratagene) as described previously [32]. pCAGGS-GCaMP2-actin was a gift from Karel Svoboda (Addgene plasmid # 18928; http://n2t.net/addgene:18928; Accessed date: January 2021; RRID: Addgene_18928) [33]. The Cofilin-pmCherryC1 was a gift from Christien Merrifield (Addgene plasmid # 27687; http://n2t.net/addgene:27687; Accessed date: January 2021; RRID: Addgene_27687) [34] and CMV-R-GECO1 was a gift from Robert Campbell (Addgene plasmid # 32444; http://n2t.net/addgene:32444; Accessed date: January 2021; RRID: Addgene_32444) [35].

### 2.4. Generation of Stable Cell Lines

To generate A375-GFP-PMCA4b, A375-GFP-PMCA4b-LA, and MCF-7-GFP-PMCA4b cell lines, A375 or MCF-7 cells were stably transfected with the SB-CAG-GFP-PMCA4b-CAG-puromycin or SB-CAG-GFP-PMCA4b-LA-CAG-puromycin using the protocols described previously [26]. To generate the MCF-7-Sh-PMCA4b cell line, MCF-7 cells were transfected with the PMCA4b shRNA plasmid (sc-42602-SH, Santa Cruz Biotechnology, Santa Cruz, CA, USA) using FuGENE HD transfection reagent (Promega, Madison, WI, USA) according to the manufacturer’s instructions. After 48 h, the culture medium was changed to the fresh medium containing puromycin dihydrochloride (1 μg/mL) (sc-108071, Santa Cruz Biotechnology) for selection. The medium with puromycin was changed every 2–3 days for two weeks. To confirm PMCA4b silencing the PMCA4b protein level was analyzed by Western blot.

### 2.5. Transient Transfection

A375 cell lines were seeded in 8-well Lab-Tek II chambered coverglass (Nalge Nunc International, Rochester, NY, USA). Next day, the cells were transiently transfected with one of the following plasmid DNA constructs (or in combination with one another): pmCherry-C1, mCherry-PMCA4b, mCherry-PMCA4b-DE, cofilin-pmCherryC1, GFP-PMCA4b-DE, pEGFP-actin, pCAGGS-GCAMP2-actin, and CMV-R-GECO1, as indicated in the experiment, using the FuGENE HD transfection reagent (Promega, Madison, WI, USA) according to the manufacturer’s recommendations. The next day, the culture medium was changed and cells were incubated for a further 48 h.

### 2.6. siRNA Transfection

PMCA4b was knocked down by siRNA treatment as described previously [27]. Briefly, A375-GFP-PMCA4b melanoma cells were seeded onto 8-well Lab-Tek II chambered coverglass (Nunc). Next day, cells were transfected with ON-Target plus SMARTpool PMCA4b (ATP2B4) siRNA (50 nM, cat. # L-006118-00-005, Dharmacon Research Inc.) or SignalSilence^®^ control siRNA (50 nM, Cell Signaling Technology, Danvers, MA, USA, cat. #6568S) using the DharmaFECT 1 transfection reagent (Dharmacon Research Inc., Cambridge, UK) according to the manufacturer’s protocol. After 24-h transfection, the medium was changed, and the cells were incubated for an additional 48 h.

### 2.7. Cell Morphology Analysis

A375, A375-GFP-PMCA4b, and A375-GFP-PMCA4b-LA cells were cultured in a 6-well plate. Phase-contrast microscope (Olympus, Japan) images were taken either after overnight cell culture (low confluence) at 10× magnification or after a 48-h cell culture period (high confluence) at 4× magnification, as stated in the figure legends. The general morphology of the cells, including area and circularity, was analyzed by applying a black mask to display the contour of the cells using ImageJ software, v1.42q (National Institutes of Health, Bethesda, MD, USA).

### 2.8. Nearest Neighbour Distance Analysis

A375-GFP, A375-GFP-PMCA4b, A375-GFP-PMCA4b-LA, MCF-7, MCF-7-GFP-PMCA4b, and MCF-7-Sh-PMCA4b cells were cultured in a 6-well plate for 48 h. Phase-contrast microscope images were taken and cell centers were obtained by using the “Particle analysis” function of the ImageJ software. Nearest neighbor distance analysis was performed on binary images of cell centers using the graph plugin of ImageJ.

### 2.9. Non-Directional Cell Motility Assay

A375, A375-GFP-PMCA4b, and A375-GFP-PMCA4b-LA cells were cultured in 96-well plates. Next day, the nuclei of the cells were stained with 0.1 μM Hoechst 33342 for 1 h. The fluorescence signals for Hoechst and GFP were acquired automatically every 30 min for 24-h at 37 °C and 5% CO_2_ using the ImageXpress Micro XL (Molecular Devices, Sunnyvale, CA, USA) high content screening system using a Nikon CFI Super Plan Fluor ELWD ADM 10× objective. For motility analysis, we used the multidimensional motion analysis module of MetaXpress High Content Image Acquisition and Analysis Software Version 5.3., Molecular Devices, Sunnyvale, CA, USA as described previously [28]. The time-lapse video demonstrating single cell motility was created from the images taken from the measurements.

### 2.10. Directional Cell Migration Assay

The Boyden chamber assay was performed as described previously [27]. Briefly, A375-GFP, A375-GFP-PMCA4b, and A375-GFP-PMCA4b-LA cells were seeded on the upper chamber of a 48-well Boyden Chamber device (Neuro probe, Gaithersburg, MD, USA) and incubated for 3 h at 37 °C. During this period, cells migrate through the 10 µm thick membrane (Whatman, Merck KGaA, Darmstadt, Germany) toward the fibronectin attractant (100 µg/mL, Merck KGaA, Darmstadt, Germany) at the lower chamber. At the end of the incubation period the membrane was removed and cells were scrapped from the upper side of the filter while at its lower side cells were fixed and stained with Toluidine blue. Light microscope images (6 images) were captured using a 10× objective lens and the number of cells migrated through the filter were counted.

### 2.11. Immunofluorescence Microscopy

A375, A375-GFP-PMCA4b, A375-GFP-PMCA4b-LA, MCF-7, MCF-7-GFP-PMCA4b, and MCF7-Sh-PMCA4b cells were seeded in 8-well Lab-Tek II chambered coverglass (Nunc) and cultured for 48 h. In some experiments, A375 cells and A375-GFP-PMCA4b cells were transiently transfected with Cofilin-pmCherryC1 or cotransfected with GFP-PMCA4b-DE in A375 cells. 48 h after transfection, cells were fixed with 4% paraformaldehyde (PFA), washed two times with PBS and then the nuclei were stained with DAPI.

For F-actin staining the cells were fixed and permeabilized with 0.1% Triton X-100 for 10 min and washed with PBS followed by incubation with Phalloidin-TRITC (Sigma-Aldrich) (1:1000) for 20 min. After that, cells were washed and kept in PBS.

For vinculin immunostaining, the cells were fixed and permeabilized as above and incubated in the blocking buffer (PBS containing 2 mg/mL bovine serum albumin, 1% fish gelatin, 0.1% Triton-X 100, 5% goat serum) for 1 h at room temperature (RT) followed by incubation with the rabbit monoclonal antivinculin antibody (1:100, ThermoFisher scientific, cat. # 700062) for 1 h at RT. After three washes with PBS, cells were incubated with AlexaFlour-647 conjugated anti-rabbit IgG (Invitrogen, Waltham, MA, USA) as a secondary antibody for 1 h at RT. Then cells were washed three times with PBS followed by staining with Phalloidin-TRITC for 30 min at RT. For nuclear staining, cells were incubated with DAPI for 10 min, washed, and kept in PBS.

To study the effect of Ca^2+^ influx on the actin cytoskeleton, A375 and A375-GFP-PMCA4b cells were seeded in 8-well Lab-Tek II chambered coverglass (Nunc). Next day, A375 cells were transiently transfected with GFP-PMCA4b-DE plasmid and cultured for 48 h. Ca^2+^ influx was initiated by the addition of 2 µM A23187 in HBSS buffer containing 2 mM Ca^2+^ (20 mM HEPES, pH 7.4, 2 mM CaCl_2_, 0.9 mM MgCl_2_) for 10 min at 37 °C. For positive and negative controls, cells were treated with 2.5 µM cytochalasin D (cytD) or with 2 µM A23187 in HBSS buffer without Ca^2+^ (in the presence of 100 µM EGTA), respectively. Cells were fixed with 4% PFA for 10 min and washed three times with PBS. Labeling with Phalloidin-TRITC is as described previously. Morphological parameters of the cells including area and circularity were analyzed using the ImageJ software.

In all experiments, confocal microscopy images were taken by Zeiss LSM710 or LSM800 confocal laser scanning microscopes using a Plan-Apochromat 40× (N.A. = 1.4) oil immersion objective (Zeiss, Oberkochen, Germany). Blue, green, red, and far red fluorescence images were sequentially acquired at 405, 488, 543, and 633 nm excitations, respectively.

### 2.12. Live-Cell Imaging

A375 cells were cultured overnight in 8-well Lab-Tek II chambered coverglass (Nunc). The next day, the cells were transfected with a GFP-actin plasmid in combination of one of the following plasmids: pmCherry-C1, mCherry-PMCA4b, or mcherry-PMCA4b-DE. After a 48-h incubation, the cells were washed twice with HBSS buffer before live-cell imaging. Experiments were initiated by the addition of (1) 2 mM A23187, (2) 2.5 µM cytD (positive control), or (3) 2 µM A23187 in HBSS buffer without Ca^2+^ (in the presence of 100 µM EGTA) (negative control). Treatments lasted for 10 min at 37 °C. Live-cell imaging was performed by acquiring Z-stack images every 15 s in both green and red channels at 488 and 561 nm excitation, respectively, using 100× 1.4 N.A. oil immersion objective of a Carl Zeiss Cell Observer SD microscope equipped with a Yokogawa CSU-X1 spinning-disk confocal module (Zeiss, Germany). For data analysis, 3D images were generated at time zero, 5 and 10 min and a video with one Z-stack was created for each experiment using ZEN 2.3 (blue edition) software, Carl Zeiss Microscopy GmbH, Jena, Germany. The Kymograph (space versus time), circulatory and area calculations relative to the zero time for each cell was analyzed using the ImageJ software.

### 2.13. Fluorescence Recovery after Photobleaching (FRAP)

A375 cells were cultured in 8-well Lab-Tek II chambered coverglass (Nunc). After overnight culture, cells were transiently transfected with plasmid constructs as described previously. For FRAP experiment, culture medium was changed with phenol free complete DMEM media supplemented with 25 mM HEPES (Gibco) and cells were kept at 37 °C. For simultaneous dual detection of GFP and mCherry fluorescence signals, 488 nm and 546 nm solid state lasers of a Carl Zeiss Cell Observer SD microscope equipped with a Yokogawa CSU-X1 spinning-disk confocal module were used. Differential interference contrast (DIC) images were taken at the end of the experiment. A defined region of interest (ROI) was drawn at three different cell parts: cell connections, cell free edge, and ruffles (lamellipodia). GFP signal was photobleached using a 488 nm bleaching laser at 20–40% intensity (RAPP UGA-42 Firefly 2L system) with a 40× 1.4 N.A. oil objective. Live cell imaging was carried out with images acquired every 0.2 s over 120 s time interval. Mean fluorescence intensity of a ROI, non-bleached region and background were analyzed using the ImageJ software. Data were imported into Microsoft Excel software 2016 (Microsoft Corporation by Impressa systems, Santa Rosa, CA, USA) and relative GFP-actin fluorescence intensity was calculated as follows: background intensity was subtracted from every ROI and at every time point, then the resulting ROI intensities were divided by a reference area intensity taken from a surrounding non-bleached cell. Post-bleach intensities were normalized to the mean of the first 10 prebleach time points. For FRAP analysis, the first 90 s of the post-bleach data was inserted into GraphPad Prism software v5.01 (GraphPad Software Inc., La Jolla, CA, USA) and non-linear regression analysis was used on the post-bleach sections to calculate the mobile fraction and half time of FRAP recovery curve (t_1/2_).

### 2.14. Ca^2+^ Signal Measurements

To detect near the actin Ca^2+^ signal, A375 cells were cultured overnight in 8-well Lab-Tek II chambered coverglass (Nunc). The next day, cells were transfected with pCAGGS-GCAMP2-actin together with one of the following plasmids: mCherry-PMCA4b, or mCherry-PMCA4b-DE and cultured for 48 h. Then cells were washed and media were replaced with HBSS buffer. Calcium influx was initiated by the addition of 2 μM A23187 for 10 min at 37 °C. Live cell imaging was performed by detecting both GCAMP2 and mCherry signals in every 15 s using the spinning-disk confocal microscope specified above and 100× 1.4 N.A. oil immersion objective. For data analysis, images were taken before, during (peak) and at 7-min after the addition of A23187. Videos were created using ZEN 2.3 (blue edition) software. Cells were analyzed for near actin Ca^2+^ signal using the ImageJ software. Relative fluorescence intensities (F/F0) were calculated with the GraphPad Prism software v5.01.

To test the distribution of basal cytosolic Ca^2+^ concentration, A375, A375-GFP-PMCA4b, and A375-GFP-PMCA4b-LA cells were seeded onto 8-well Lab Tek II chambered coverglass (Nunc). The next day, the cells were transiently transfected with CMV-R-GECO1 plasmid and cultured for 48 h. Confocal microscope images were taken using confocal laser scanning microscope, Zeiss LSM710 with 63× oil immersion objective (Zeiss, Germany). Line plot analysis of the fluorescence signal was performed using the ImageJ software.

### 2.15. Western Blot Analysis

A375, A375-GFP-PMCA4b, A375-GFP-PMCA4b-LA, MCF-7, MCF-7-GFP-4b, and MCF-7-Sh-PMCA4b cells were cultured in a 6-well plate for 48 h. The total protein content of the cells was precipitated with 6% TCA. Samples were separated by using 10% or 15% acrylamide gels, as appropriate, and electroblotted onto PVDF membranes (Biorad, Hercules, CA, USA), as described previously [28].

Blots were immunostained with the following rabbit monoclonal primary antibodies: antivinculin (1:100, ThermoFisher scientific, cat. # 700062), anti-P-cofilin (Ser3) (1:1000, Cell Signaling Technology, Danvers, MA, USA, cat. # 77G2), rabbit polyclonal antibody: anti-β-tubulin (1:1000, Abcam, cat. # ab6046), anti-PMCA1 (1:1000, Affinity BioReagents, cat. # PA1-914), mouse monoclonal antibodies: anti-PMCA4 (JA9, 1:1000, Sigma-Aldrich, cat. # P1494), anti-NA^+^/K^+^ ATPase (1:2000, Enzo Life Sciences, cat. # BML-SA247), and chicken polyclonal antibody: anti-GFP (1:5000, Aves, GFP-1020). Horseradish peroxidase-conjugated anti-rabbit, anti-mouse, or anti-chicken secondary antibodies were used for detection (Jackson ImmunoResearch, dilution 1: 10,000) and were visualized with Pierce ECL Western Blotting Substrate (Thermo Scientific). The ImageJ software was used for densitometry analysis.

### 2.16. Statistical Analysis

For the stress fiber and lamellipodia formation analysis, a Chi square test while for Western blots, an unpaired *t*-test was used. For FRAP analysis, area and circulatory calculations, the differences between the control and the experimental groups were determined by a one-way analysis of variance (ANOVA) followed by Dunnett’s multiple comparison post-hoc test, or unpaired *t*-test for two groups comparison. For circularity and area data versus time for the three groups, two-way ANOVA followed with the Bonferroni post-hoc test was used. The difference was considered significant at *p* < 0.05. The asterisks *, **, and *** denote values <0.05, <0.01, and <0.001, respectively.

## 3. Results

### 3.1. Proper Trafficking of PMCA4b Is Required to Change A375 Melanoma Cell Morphology, and Migration.

**Figure 1:**
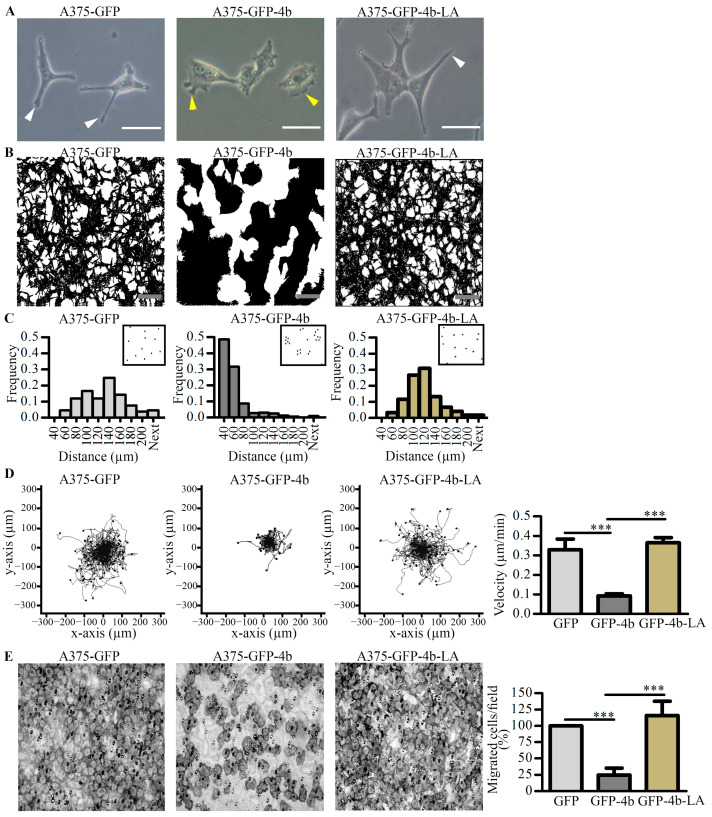
PMCA4b but not the trafficking mutant PMCA4b-LA changed shape, culture morphology, and migration-type of A375 cells. (A+B) A375-GFP, A375-GFP-PMCA4b, and A375-GFP-PMCA4b-LA cells were cultured in a 6-well plate. After overnight attachment and at 80% confluency images were taken using a phase-contrast microscope. Cell culture morphology was highlighted by applying a black mask to display the contour of the cells. White and yellow arrowheads show protrusions and lamellipodia, respectively. Scale bar, (**A**) 10 µm and (**B**) 50 µm. (**C**) After 48 h in culture, phase-contrast microscopy images were taken. Cell centers were determined and nearest neighbor distances were calculated from the binary images. Insets show the center of cells, as dots. (**D**) Cells were cultured in a 96-well plate and stained with Hoechst 33342. Migratory activity of the cells was followed by recording Hoechst and GFP signals by automated fluorescence microscopy for 24 h. Single cell trajectories of A375-GFP (*n* = 130), A375-GFP-PMCA4b (*n* = 77), and A375-GFP-PMCA4b-LA (*n* = 101) with the starting position of each trajectory translated to the origin of the plot are shown. Mean velocity ± S.D was determined from single cell trajectories (A375-GFP (*n* = 645), A375-PMCA4b (*n* = 941), and A375-PMCA4b-LA (*n* = 990) of 4–5 independent measurements. (**E**) For directional cell migration Boyden chamber assay was performed. Cells were seeded into the upper chamber and left to migrate for 3 h through the filter membrane towards the fibronectin at the lower chamber. Cells at the bottom of the filter membrane were fixed and stained with Toluidine blue. The number of migrated cells from six field of view was counted. Data show the means (% relative to the control) ± S.E.M from three independent experiments (*** *p* < 0.001).

Previously, we demonstrated that overexpression of PMCA4b induced a profound change in the shape and motility of A375 melanoma cells [26]. Endocytic trafficking has been suggested to regulate both cell shape and motility in a variety of cell models [36,37,38]. Our laboratory has identified a di-leucine-like ^1167^LLL internalization signal at the C-tail of PMCA4b. Mutation of these leucines to alanines resulted in a trafficking mutant (PMCA4b-LA), which has been characterized by having impaired endocytosis and hence high cell surface expression [31]. To test if endocytic trafficking of PMCA4b was essential for the distinct migratory and cell shape character of the melanoma cells, we compared shape and migration of GFP or GFP-PMCA4b expressing cells to those of the trafficking mutant GFP-PMCA4b-LA (Figure 1, Figures S1A and S8). At the single cell level, GFP-PMCA4b expression resulted in transition from a spindle-shaped character with three to four protrusions per cell to a polarized mesenchymal appearance with a typical asymmetric lamellipodial architecture, similarly to that shown previously [26]. In contrast, the A375-GFP-PMCA4b-LA cells retained the spindle-shaped character of the control A375-GFP cells (Figure 1A), and no significant change in area and circularity parameters could be detected (Figure S1B). In subconfluent cell cultures, the A375-GFP-PMCA4b cells formed clusters, whereas the A375-GFP-PMCA4b-LA cells showed scattered distribution similarly to that seen in the A375-GFP cells (Figure 1B). The nearest neighbor distribution histogram of A375-GFP-PMCA4b cells was shifted to the left as compared to the control or to the A375-GFP-PMCA4b-LA cells suggesting closer contact between the PMCA4b expressing cells in subconfluent culture (Figure 1C).

A375 cells are a highly motile type of melanoma cells as they show dynamic protrusion and retraction activities with constantly changing direction of displacement. PMCA4b expression changed dramatically this character to a slow-moving mesenchymal-type with intense lamellipodia membrane ruffling and shorter displacement over time while the cells expressing the trafficking mutant A375-GFP-PMCA4b-LA, remained highly motile (Figure 1D and Figure S2, Videos S1 and S2). Similar results were obtained using a directional migration assay, in which the cells moved through the filter of a Boyden Chamber towards fibronectin as an attractant. Again, PMCA4b expression inhibited migration of the cells while expression of the mutant PMCA4b-LA had no effect (Figure 1E). Taken together, our data suggest that proper trafficking of PMCA4b was crucial for determining the shape and migratory behavior of these BRAF mutant melanoma cells.

### 3.2. PMCA4b Induces Actin Cytoskeleton Remodeling in A375 Melanoma Cells

**Figure 2:**
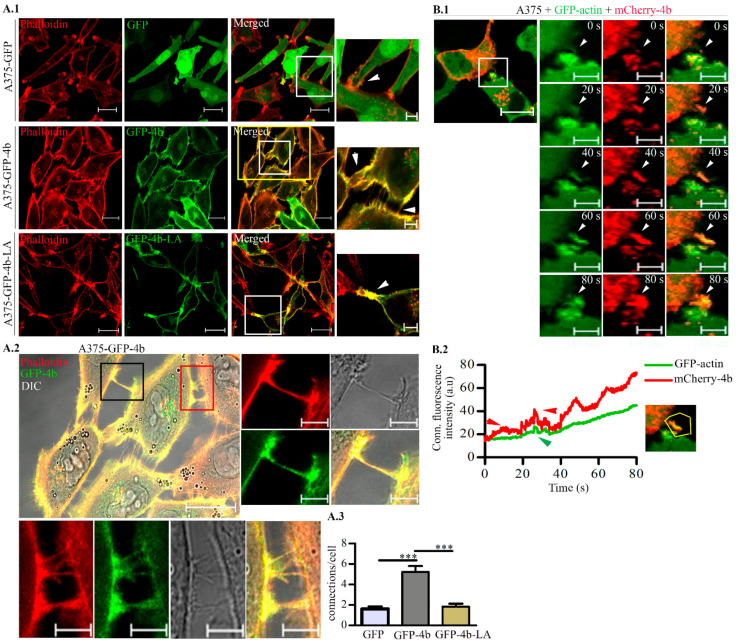
PMCA4b but not the trafficking mutant PMCA4b-LA increased cell–cell connections between A375 cells. (**A.1**) Confocal microscopy images of A375-GFP, A375-GFP-PMCA4b, and A375-GFP-PMCA4b-LA cells labeled with Phalloidin-TRITC. Scale bar, 20 µm. Insets show images with higher magnification of field marked with white rectangles; arrowhead indicate cell–cell connection. Scale bar, 5 µm. (**A.2**) High magnification DIC and fluorescence images of A375-GFP-PMCA4b cells are taken from the field marked with the yellow rectangle in (**A.1**). Scale bar, 20 µm. The black and red insets show images for the intercellular connections formed between cells. Scale bar, 5 µm (**A.3**) The graph represents the mean number of connections/cell for 12–13 cell. (**B.1**) Live cell imaging of A375 cells transiently expressing GFP-actin and mCherry-PMCA4b recorded by spinning-disc confocal microscopy. GFP and mCherry signals were recorded every 0.2 s for 180 s at 37 °C. Scale bar, 20 µm. Insets show the formation of connection between two cells with higher magnification at different times. Scale bar, 5 µm. (**B.2**) The graph depicts the time courses of GFP-actin and mCherry-PMCA4b signals determined in the region of interest (ROI) drawn around a newly forming connection (yellow polygon). Arrowheads indicate the increased signal (*** *p* < 0.001).

Remodeling of the actin cytoskeleton plays a role in determining cell shape and migration [39]. One of the key regulators of actin dynamics is Ca^2+^ that acts through a variety of Ca^2+^-dependent regulatory mechanisms [8,9]. Since PMCAs are considered as important regulators of intracellular Ca^2+^ concentration, we labeled A375 cells with Phalloidin-TRITC and studied the role of PMCA4b in F-actin organization with confocal laser scanning microscopy. We found that the A375-GFP-PMCA4b cells displayed an increased number of intercellular connections compared to the A375-GFP-PMCA4b-LA or A375-GFP cells where only few (one or two) connections could be detected (Figure 2A). Live-cell imaging experiments on mCherry-PMCA4b and GFP-actin coexpressing cells show that the mCherry-PMCA4b signal was followed by the thickening GFP-actin-based protrusions during the formation of cell–cell connections (Figure 2B and Video S3).

**Figure 3:**
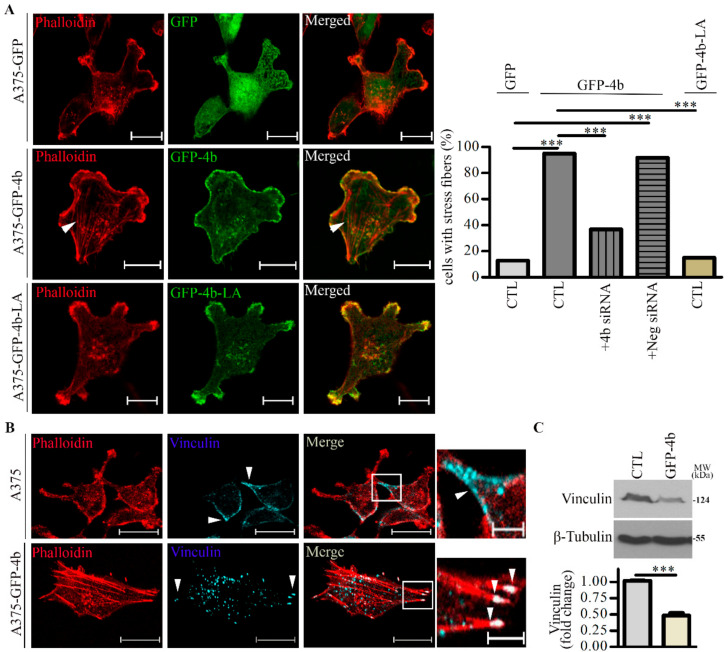
Lamellipodia and stress fiber formation are increased in A375 cells expressing GFP-PMCA4b but not in those expressing GFP-PMCA4b-LA. (**A**) Confocal microscopy images of A375-GFP, A375-GFP-PMCA4b, and A375-GFP-PMCA4b-LA cells labeled with Phalloidin-TRITC. Arrowheads show stress fibers. Scale bar, 20 µm. The fractions of stress fiber-positive cells in A375-GFP (*n* = 55), A375-GFP-PMCA4b-LA (*n* = 72), A375-GFP-PMCA4b (*n* = 60) ± PMCA4b siRNA (*n* = 68), and negative siRNA (*n* = 49) cultures were determined. siRNA confocal microscopy images are presented in Figure S3A. Relative abundance of cells with stress fibers is indicated by the bar graph. Confocal sections were taken from the bottom of the cells to show the stress fibers. (**B**) Confocal microscopy images of A375 and A375-GFP-PMCA4b cells immunostained with vinculin and labeled with Phalloidin-TRITC. Scale bar, 20 µm. Insets show part of the cells with higher magnification. Arrowheads show the differential localization of vinculin. Scale bar, 5 µm. (**C**) A375 and A375-GFP-PMCA4b cells were cultured in a 6-well plate for 48 h. Vinculin protein expression from total cell lysate was analyzed by Western blotting. β-tubulin was used as a loading control. Data represent mean ± S.E.M from three independent experiments (*** *p* < 0.001).

Figure 3A shows that A375-GFP-PMCA4b cells formed lamellipodia and actin stress fiber bundles in contrast to the A375-GFP-PMCA4b-LA and A375-GFP cells, which have more actin-rich membrane protrusions with significantly less stress fibers at the cell bottom. The graph indicates that GFP-PMCA4b expression increased the number of cells with stress fibers by more than 80% when compared to the GFP or the GFP-PMCA4b-LA mutant expressing cells. Treatment of the A375-GFP-PMCA4b cells with PMCA4b siRNA significantly reduced the number of cells with stress fibers confirming the role of PMCA4b in stress fiber formation (Figure S3A).

It has been demonstrated that free cytosolic Ca^2+^ concentration has a role in regulating focal adhesion turnover [40,41]. Therefore, we stained A375 and A375-GFP-PMCA4b cells for vinculin and F-actin, and found that vinculin dots clustered near the cell periphery towards the protrusions of the control A375-GFP cells, which was different from the punctate pattern at the focal adhesion sites of the stress fibers in the A375-GFP-PMCA4b cells (Figure 3B). This change was accompanied by decreased expression of vinculin in the A375-GFP-PMCA4b cells (Figure 3C). These data suggest stronger adhesion of the PMCA4b overexpressing cells to the substrate than the cells without PMCA4b.

### 3.3. A Functional PMCA4b Pump Is Needed for Actin Cytoskeleton Remodeling

**Figure 4:**
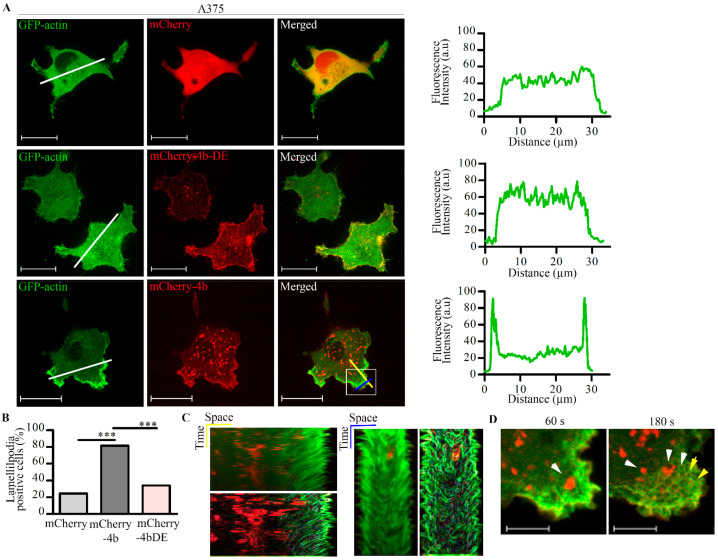
PMCA4b activity is necessary for lamellipodia formation in A375 melanoma cells. (**A**) A375 cells were transfected with GFP-actin together with one of the following plasmids: pmCherry-C1, mCherry-PMCA4b, mCherry-PMCA4b-DE, and cultured for 48 h. Confocal microscopy images of lamellipodia are shown. Scale bar, 20 µm. Graphs represent the GFP-actin intensity profile (green) for the line (white) drawn along the cell as indicated in the confocal image. Confocal sections were taken in the middle to visualize lamellipodia formation. (**B**) The graph shows the fraction of lamellipodia-positive cells in cultures transfected with GFP-actin and mCherry, mCherry-PMCA4b, or mCherry-PMCA4b-DE. (**C**,**D**) Live-cell imaging of cells expressing GFP-actin and mCherry-PMCA4b. Z-stacks of images were taken every 5 s for 5 min at 37 °C using spinning-disc confocal microscopy. (**C**) Kymographs were generated along the lines (yellow and blue) of the lamellipodia of the mCherry-PMCA4b cell shown in the image in (**A**), using the ImageJ software. Fine edges of ruffles are shown. (**D**) Magnified part of the lamellipodia from the same cell shown in (**A**). White arrowheads show mCherry-PMCA4b positive vesicles and the yellow arrowheads show GFP-actin retrograde flow. Scale bar, 5 µm (*** *p* < 0.001).

To test if PMCA4b function is required for actin cytoskeleton remodeling we used the non-functional mutant pump mCherry-PMCA4b-DE, in which we introduced an aspartate-to-glutamate substitution at position 672 [32]. Since this mutant cannot transport Ca^2+^, it helped to dissect further the functional role of PMCA4b in actin cytoskeleton remodeling. In order to study this, we transiently expressed wild-type or mutant mCherry-tagged PMCA4b pump together with GFP-actin in A375 cells. It is worth noting that neither GFP nor mCherry tags affected PMCA4b activity, as described previously [32,42]. In the mCherry-PMCA4b expressing cells, GFP-actin concentrated to the lamellipodia at the cell front (Figure 4A) and formed stress fibers at the cell bottom (Figure S3B) as expected. In the non-functional mCherry-PMCA4b-DE expressing cells, however, the GFP-actin signal distributed evenly throughout the cell, and a significant reduction in stress fiber and lamellipodia formation was detected (Figure 4A,B and Figure S3B). A kymograph (Figure 4C) along the lines drawn across the lamellipodia of a mCherry-PMCA4b expressing cell shows highly polymerized actin, and high frequency of lamellipodial ruffling and retraction activity. We found mCherry-PMCA4b positive vesicles moving toward the edge of the lamellipodia and backwards with the GFP-actin retrograde flow (Figure 4D and Video S4). Together, these findings suggest that PMCA4b activity was essential for both stress fiber and lamellipodia formation, and that intense PMCA4b trafficking accompanied membrane ruffling at the lamellipodia.

### 3.4. Silencing PMCA4b Expression Decreases the Number of Cells with Stress Fibers and Changes Cell Culture Morphology of MCF-7 Breast Cancer Cells

**Figure 5:**
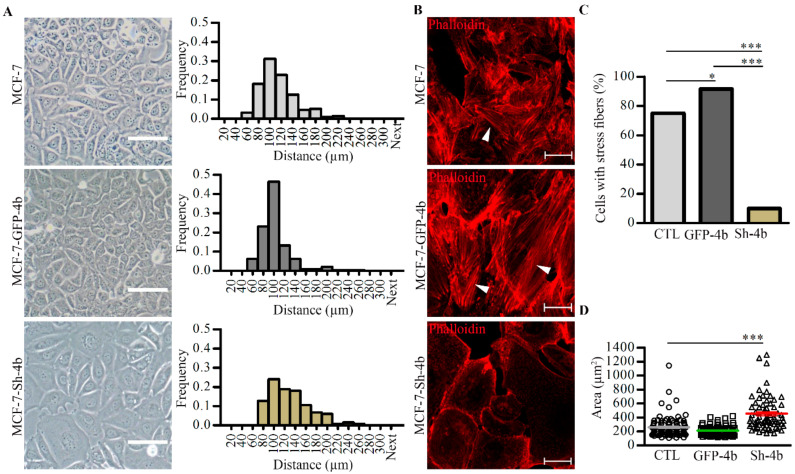
PMCA4b silencing in MCF-7 cells changed cell culture morphology and resulted in a dramatic loss of stress fibers. (**A**) Phase contrast images of MCF-7 cells, GFP-PMCA4b (MCF-7-GFP-4b), or shRNA of PMCA4b (MCF-7-Sh-4b). Scale bar, 50 µm. The distance between nearest neighbors was determined using the binary images of cell centers. (**B**) F-actin in the three cell types was labeled with Phalloidin-TRITC. Arrowheads show stress fibers. Scale bar, 20 µm. The fractions of stress fiber-positive cells were determined (*n* = 48, 83, and 100, respectively) (**C**) and the area of individual cells of the phase contrast images was calculated (*n* = 100, 88, and 70, respectively) (**D**). Bars represent the mean values for each cell type (* *p* < 0.05, *** *p* < 0.001).

To confirm that the effect of PMCA4b on actin rearrangement is not cell type-specific; we used the estrogen receptor positive (ER+) luminal type of breast cancer cell line MCF-7. We showed that PMCA4b expression is relatively low in these cells that can be greatly upregulated with histone deacetylase (HDAC) inhibitors. Since we surmised that HDAC inhibitors could interfere with our studies, we either stably expressed or silenced PMCA4b in MCF-7 breast cancer cells. The Western blots in Figures S4 and S8 show that MCF-7 cells express PMCA4b endogenously and that silencing diminished its expression almost completely. In accordance with the relative PMCA4b abundance, changes in cell culture morphology were observed. Nearest neighbor distance histogram analysis shown in Figure 5A indicated that MCF-7-GFP-PMCA4b cells were located closer to each other than the parental or the PMCA4b silenced cells. In addition, PMCA4b silencing induced a dramatic—more than 60%—loss of stress fibers (Figure 5B,C) and increased significantly the area of individual cells (1.75×) (Figure 5A,D) underlying the importance of PMCA4b in actin cytoskeleton remodeling, and consequently changes in cell shape and cell culture morphology.

### 3.5. PMCA4b Does Not Affect F-Actin Recovery after Photobleaching

**Figure 6:**
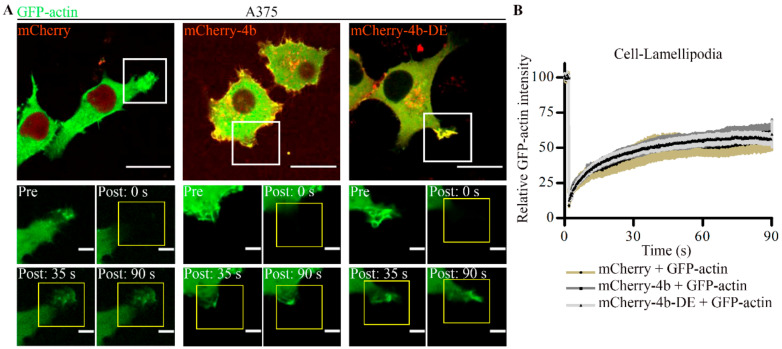
PMCA4b expression does not affect F-actin turnover in A375 cells. (**A**) A375 cells were transfected with GFP-actin together with one of the following plasmids, pmCherry-C1, mCherry-PMCA4b, mCherry-PMCA4b-DE, and cultured for 48 h. Media were changed to phenol free DMEM and cells were kept at 37 °C. GFP-actin was photobleached at the ruffles (mCherry: *n* = 8, mCherry-4b: *n* = 13, mCherry-4b-DE: *n* = 23) using spinning-disc confocal microscopy. GFP signal was recorded every 0.2 s for 90 s. Scale bar, 20 µm. Insets show magnified lamellipodia before (pre) and after (post) photobleaching at different time points. Scale bar, 2 µm. (**B**) Relative GFP-actin fluorescence intensity changes over time. Data represent mean ± S.E.M for three independent experiments.

Fluorescence recovery after photobleaching (FRAP) is often used to study actin cytoskeleton dynamics [43]. From FRAP analysis both the mobile fraction and turnover rate (t_1/2_) of F-actin can be determined. To reveal whether changes in cell morphology was a result of F-actin dynamics, we performed FRAP analysis in cells transfected with GFP-actin and mCherry, mCherry-PMCA4b, or mCherry-PMCA4b-DE. FRAP was performed at three different parts of the cells: at the cell-free edge, at the cell–cell contacts (Figure S5), and at the lamellipodia (Figure 6). We found that PMCA4b expression did not affect either the mobile fraction or the recovery rate of F-actin at any of these locations. Although the mCherry-PMCA4b expressing cells showed slightly faster recovery rate (lower t_1/2_) at the lamellipodia than the parental cells, the difference was not significant (Figure 6B and Figure S5B) suggesting that F-actin assembly was not affected by PMCA4b.

### 3.6. PMCA4b Inhibits Ca^2+^ Induced F-Actin Depolymerization

**Figure 7:**
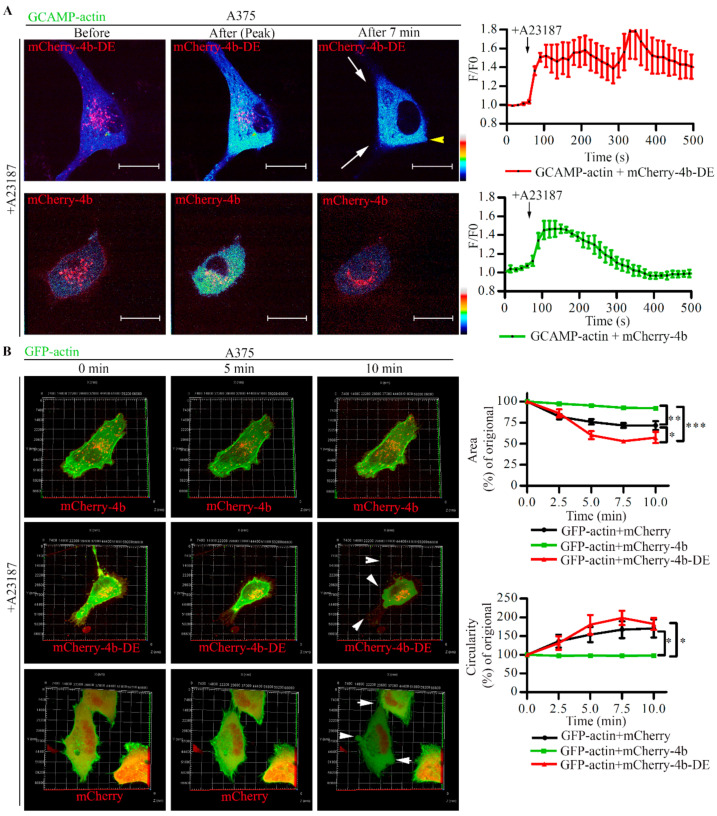
PMCA4b protects F-actin from Ca^2+^-induced degradation. (**A**) Near actin Ca^2+^ signal was initiated in A375 cells transiently expressing GCAMP2-actin together with mCherry-PMCA4b or mCherry-PMCA4b-DE by the addition of 2 µM A23187. GCAMP2 and mCherry signals were recorded every 15 s for 10 min using a spinning-disc confocal microscopy. Confocal microscopy images show cells before, at the peak and 7 min after the addition of A23187, as indicated. Arrowheads show GCAMP2-actin signal retraction. Scale bar, 20 µm. Graphs represent fluorescence intensity values (F/F0) of the cell shown in (**A**). Error bars represent S.E.M obtained from two independent experiments (two cells analyzed in each, two ROIs per cell). Arrows indicate the addition of A23187. (**B**) 2 µM A23187 was added to A375 cells expressing GFP-actin together with pmCherry-C1, mCherry-PMCA4b, or mCherry-PMCA4b-DE, and Z-stack images of GFP and mCherry signals were recorded every 15 s for 10 min using a spinning-disc confocal microscope. Three-dimensional confocal images were created by the ZEN 2.3 (blue edition) software, and presented at 0, 5, and 10 min. Arrowheads indicate changes in cell shape. Area and circularity parameters for A375 cells transfected with the same plasmid combinations (*n* = 2–3 each) were analyzed by the ImageJ software. Data represent mean ± S.E.M. Significance is calculated for the 10-min time points (* *p* < 0.05, ** *p* < 0.01, *** *p* < 0.001).

Several studies have demonstrated that high cytosolic Ca^2+^ concentration can induce F-actin depolymerization [6,44,45,46]. To investigate if PMCA4b is able to protect the actin cytoskeleton from Ca^2+^ overload, we used A375 cells expressing GCaMP2- or GFP-actin together with mCherry, mCherry-PMCA4b, or the non-functional mutant mCherry-PMCA4b-DE. Cells were treated with the Ca^2+^ ionophore A23187 to allow Ca^2+^ influx. As expected, A23187 induced a sustained increase in GCaMP2-actin fluorescence in cells expressing the non-functional mutant PMCA4b-DE, while the fluorescence returned relatively quickly to the basal level in cells expressing the wild-type pump protein (Figure 7A). In correlation with the sustained increase in near-actin Ca^2+^ concentration, a dramatic loss of cell protrusions, intense membrane blebbing, and cell shrinkage was observed in the mCherry or the mCherry-PMCA4b-DE expressing cells (Figure 7B, see also Video S5). In correlation with these changes, a significant increase in circularity (1.64 and 1.83 fold) and a decrease in area (28.4% and 42.7%) were detected in the control and mutant pump expressing cells, respectively. In contrast, no change in these parameters was observed in the wild-type PMCA4b expressing cells displaying intact stress fibers at the cell bottom after treatment (Figure 7B). A kymograph in Figure S6A shows GFP-actin collapse at the cell periphery both in the mCherry and the mCherry-PMCA4b-DE expressing cells, while the mCherry-PMCA4b cells retained their original shape.

**Figure 8:**
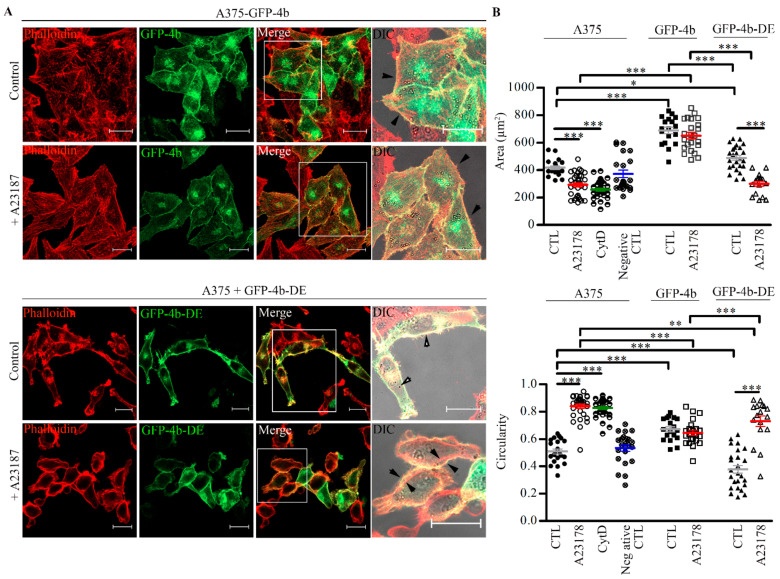
A375-GFP-PMCA4b but not the parental or the non-functional mutant expressing PMCA4b-DE cells maintain shape after Ca^2+^ overload. (**A**) A375-GFP-PMCA4b cells or A375 cells transiently expressing GFP-PMCA4b-DE were treated with 2 µM A23187 in HBSS buffer containing 2 µM Ca^2+^ for 10 min at 37 °C. Confocal and DIC microscopy images were taken after labeling with Phalloidin-TRITC. Scale bar, 20 µm. Right images show cells with higher magnification, scale bar, 20 µm. Arrowheads show the position of actin in relation to the cell periphery. (**B**) Area and circularity parameters were analyzed for each cell type (*n* = 17–33) by ImageJ software. The mean ± S.E.M values of data are presented as a scatter plot. Confocal microscopy images of cells incubated in the presence or absence of cytochalasin D (CytD) or A23187 are shown in Figure S7 (* *p* < 0.05, ** *p* < 0.01, *** *p* < 0.001).

Similar results were obtained when the endogenous F-actin was labeled with Phalloidin-TRITC. Treatment of the parental and GFP-PMCA4b-DE expressing cells with A23187 led to cell shrinkage, rounding, and intense membrane blebbing, while the A375-GFP-PMCA4b cells did not show any of these changes even after a relatively long exposure to the ionophore (Figure 8 and Figure S7A). When ionophore was added in the absence of external Ca^2+^ (in the presence of EGTA), none of the cells showed change in morphology confirming the role of excess Ca^2+^ entry. As a control, the actin depolymerizing agent cytochalasin D destroyed the actin cytoskeleton independent of Ca^2+^ entry (Figures S6B and S7B, and Video S6). These results underlie the importance of the Ca^2+^ extrusion capacity of PMCA4b that can reduce near-actin Ca^2+^ concentration levels protecting the actin cytoskeleton from Ca^2+^ overload.

### 3.7. PMCA4b Induces F-Actin Rearrangement through Cofilin Relocation.

**Figure 9:**
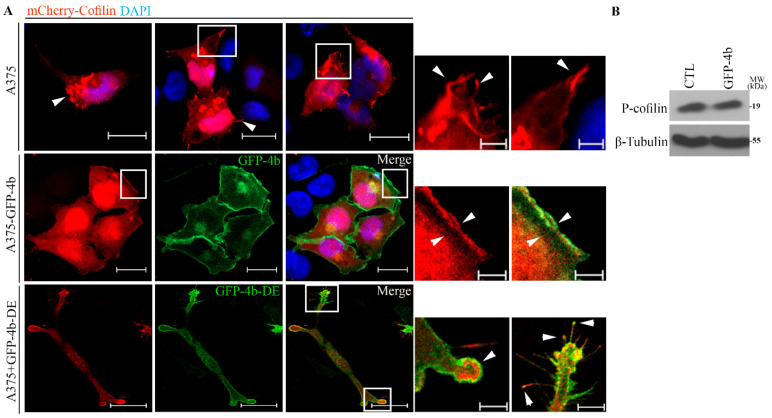
PMCA4b expression results in cofilin reorganization to the leading edge. (**A**) mCherry-Cofilin was transfected in A375 and A375-GFP-PMCA4b cells or cotransfected with GFP-PMCA4bDE in A375 cells. Confocal microscopy images were taken after nuclear staining with DAPI. Scale bar, 20 µm. Insets show a part of a cell with higher magnification. Arrowheads show the location of expressed cofilin at the leading edge in A375-GFP-PMCA4b cells and filopodia or protrusion in A375 cells and A375 expressing GFP-PMCA4bDE. Scale bar, 5 µm. (**B**) A375 and A375-GFP-PMCA4b cells were cultured in a 6-well plate for 48 h. Endogenous P-cofilin protein expression from total cell lysate was analyzed by Western blotting. β-tubulin was used as a loading control.

Several studies described the role of cofilin in actin dynamics [19,47]. Cytosolic Ca^2+^ is an important regulator of cofilin activity [21], therefore, we investigated if PMCA4b expression altered the distribution of mCherry-cofilin in A375 cells. Figure 9A shows that mCherry-cofilin localized to the protrusions of the parental and the non-functional GFP-PMCA4bDE expressing cells, while in the A375 cells expressing active GFP-PMCA4b, it localized mostly to the leading edge of the lamellipodia. One of the mechanisms that regulate cofilin activity is its phosphorylation at serine 3. Therefore, we tested P-cofilin at the protein level, but no differences were detected between the parental and A375-GFP-PMCA4b cells (Figure 9B). These results suggest that cofilin relocalization to the leading edge rather than changes in its overall activity contributed to the actin cytoskeleton rearrangement of PMCA4b expressing cells.

### 3.8. Proper PMCA4b Trafficking Is Essential in Managing Front-to-Rear Increasing Ca^2+^ Concentration Gradient in A375 Cells

**Figure 10:**
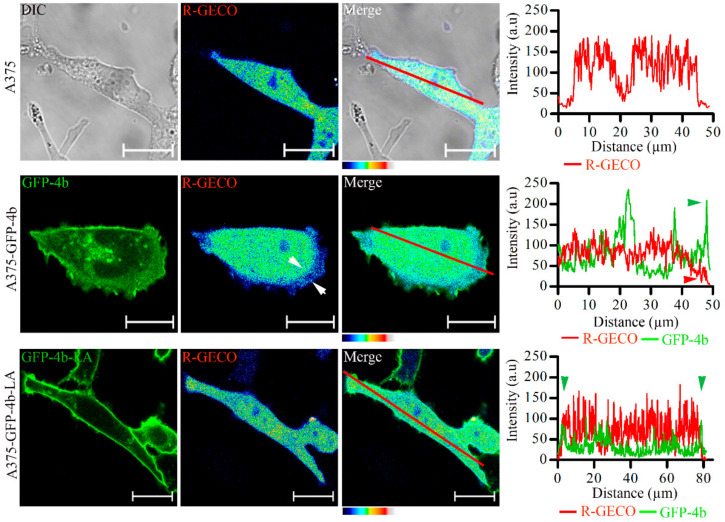
Proper trafficking of PMCA4b is needed to maintain a front-to-rear Ca^2+^ concentration gradient in A375 cells. A375, A375-GFP-PMCA4b, and A375-GFP-PMCA4b-LA cells were transfected with the CMV-R-GECO1 plasmid. Cells were fixed and confocal microscopy images were taken. Low Ca^2+^ levels are indicated by the arrowheads at the leading edge of A375-GFP-PMCA4b cells. Scale bar, 20 µm. Graphs represent GFP-PMCA4b (green) and R-GECO intensity (red) profiles for the line (red) drawn along the cells on the confocal images. Arrowheads show the GFP signal at the cell periphery. Line plots were drawn and analyzed using the ImageJ software.

A previous study suggested that polarized distribution of PMCA4b contributes to the front-to-rear Ca^2+^ concentration gradient during migration of Human Umbilical Vein Endothelial Cells (HUVECs) [48]. To investigate if PMCA4b localization to the leading edge resulted in a similar front-to-rear Ca^2+^ concentration gradient in A375 cells, we expressed the Ca^2+^ indicator R-GECO in the A375-GFP-PMCA4b or A375-GFP-PMCA4b-LA cells. Fluorescence intensities of R-GECO and GFP signals were recorded and analyzed across the lines shown on the confocal microscope images in Figure 10. The A375-GFP-PMCA4b cells displayed polarized distribution of the GFP-PMCA4b signal that was accompanied by an inverse distribution of the R-GECO signal. In contrast, the control and the trafficking mutant expressing cells displayed an even distribution of Ca^2+^ concentration across the entire cytoplasm (Figure 10). Our data suggest that PMCA4b is essentially involved in maintaining a Ca^2+^ concentration gradient increasing front-to-rear that may contribute to the actin cytoskeleton reorganization and the formation of the low-motility mesenchymal cell phenotype.

## 4. Discussion

The calcium ion is considered as a modulator of actin dynamics, and a higher intracellular Ca^2+^ concentration is found to promote cell migration [8,11]. Previously, we identified the PMCA4b Ca^2+^ pump as a putative metastatic suppressor in BRAF mutant melanoma cells [26]. Since PMCAs are considered as key regulators of cellular Ca^2+^ homeostasis, we hypothesized that the reduced migration and morphology changes observed along with the reduced metastatic activity of these melanoma cells are correlated with changes in actin dynamics.

Melanocytes in the skin through their dendrites are in close contact with the surrounding keratinocytes and while melanocytes produce melanin to protect keratinocytes from UV radiation keratinocytes tightly control the proliferation capacity of melanocytes [49]. Across the epidermis, there is an increasing Ca^2+^ gradient that decreases proliferation and induces differentiation of keratinocytes up to the stratum corneum where Ca^2+^ concentration sharply decreases. This refers to both the extracellular Ca^2+^ concentration and the amount of Ca^2+^ in the intracellular Ca^2+^ stores. During differentiation expression of Ca^2+^ regulatory molecules such as Ca^2+^ channels and the Ca^2+^ sensing receptor are gradually changing that results in altered expression of differentiation markers and desmosome formation [50]. Interestingly, melanoma cells influence the differentiation pattern of the keratinocytes in the vicinity of the tumor through the production of growth factors and cytokines that induces the hyperplasia of the epidermis [51,52]. However, the role PMCA proteins in these processes is not known.

Melanoma cells tend to have great plasticity in shifting between mesenchymal and amoeboid motility style to allow cells to invade and metastasize [53,54]. Here we demonstrate that spontaneous movement of A375-GFP-PMCA4b cells has slower polar type motility in contrast to the fast-moving parental cells with dynamic actin-rich filopodia formation. Since a previous study from our laboratory did not find any significant changes in the expression of EMT/MET markers (E-cadherin, ZEB1, snail, and vimentin) [26], we surmise that PMCA4b expression induced a switch from a fast- to a slow-moving mesenchymal cell type rather than a transition between mesenchymal/epithelial phenotypes [55].

Several studies have reported on the role of cytoplasmic free Ca^2+^ in changing cell morphology through the induction of actin cytoskeleton reorganization in a variety of cell types [56,57]. A study on pulmonary endothelial cells reported that the activation of store-operated Ca^2+^ channels (SOCs) resulted in cell shape changes and this was dependent on site-specific reorganization of the actin cytoskeleton [58]. In agreement with these findings, we show that along with the switch between motility types, PMCA4b expression induced a dramatic change in cell morphology both at the single cell and cell culture levels including cell roundness, increased formation of cell–cell connections, lamellipodia formation, and stress fibers with localized distribution of focal adhesion sites. Interestingly, similar morphology changes were seen in the MCF-7 breast cancer cells suggesting a general role of PMCA4b in cell shape determination.

Stress fibers and their associated focal adhesions are important for cells to adhere [59]. It was found that highly motile cells have few, thin, and dynamic stress fibers compared to the thick and stable stress fibers of non-motile cells [60]. It was also suggested that cell motility inhibition could be a result of slow rearrangement of stress fiber actin bundles and focal adhesion [59]. This is in good correlation with the slow motility of A375-GFP-PMCA4b cells, which have thick stable stress fiber bundles in contrast to the fast-moving parental cells, which lack stress fibers almost completely. Again, the effect of PMCA4b on stress fiber formation was not restricted to the melanoma cell type; PMCA4b silencing caused a nearly complete loss of stress fibers in the breast cancer MCF-7 cell line, as well.

High free intracellular Ca^2+^ can increase the focal adhesion turnover rate and cell motility [8]. In the current study, we found punctate localization of vinculin at focal adhesion sites in the A375-GFP-PMCA4b cells as compared to the clustered vinculin dots near the cell periphery facing towards the protrusions in the parental cells. A study [61] showed that blocking store-operated Ca^2+^ influx (SOCE) in MDA-MB-231 cells resulted in different localization of vinculin that caused slow focal adhesion turnover rate and strong cell adherence. Similarly, another study on mesenchymal-like chemoresistant IGROV1 ovarian cancer cells showed enhanced cell migration as a result of enhanced focal adhesion turnover mediated by SOCE [62]. It has been suggested that PMCA4b has the ability to decrease near-membrane Ca^2+^ concentration in response to SOCE [63] that may explain, at least partly, the enhanced focal adhesion and reduced motility of the PMCA4b expressing melanoma cells.

High expression of vinculin has been found in cancerous cells and was used as a biomarker in pancreatic cancer [64]. A study reported that electromagnetic fields enhanced cell migration in bone marrow-derived mesenchymal cells in a Ca^2+^-dependent manner by increasing the expression of several focal adhesion proteins including vinculin [65]. Using a Ca^2+^ channel blocker, vinculin expression decreased to its baseline level that reduced cell migration. Interestingly, PMCA4b expression resulted in decreased vinculin expression that might contribute to the reduced migratory activity of the PMCA4b expressing A375 melanoma cells.

Changes in the localization and trafficking of membrane proteins have been correlated to cell shape and motility [36,37,38]. In line with these findings we found that the trafficking mutant PMCA4b-LA did not affect shape, migration, and F-actin distribution of A375 cells. We showed that the characteristics of the PMCA4b-LA expressing cells resembled more that of the control cells than those of the wild-type PMCA4b expressing cells suggesting that proper localization was essential for the antimigratory behavior of the pump.

Further, we showed that expression of the non-functional PMCA4b-DE did not induce rearrangement of the actin cytoskeleton confirming that PMCA4b activity and hence, intracellular Ca^2+^ concentration played a role. Similarly, a study reported that activation of transient receptor potential melastatin 2 (TRPM2) Ca^2+^ channel by H_2_O_2_ in Hela and prostate cancer (PC)-3 cells resulted in filopodia formation, loss of stress fibers, and disassembly of focal adhesion that eventually caused an increase in cell migration [66]. Another study on highly metastatic osteosarcoma cell line U2OS reported that Ca^2+^ channel ORAI1 translocation to the leading edge was essential for formation of lamellipodia and cell directionality [67]. Our data are in good agreement with these findings and points to the importance of PMCA4b in mediating actin cytoskeleton rearrangement and cell motility through controlling cytosolic Ca^2+^ levels.

In polarized cells, actin reorganization at the lamellipodia of the leading edge directs cell migration [68]. We noticed polymerized actin at the lamellipodia occupying most of the cell front when a functional PMCA4b was expressed in contrast to the cells expressing the non-functional PMCA4b-DE mutant or the control cells where F-actin was more abundant at the pointed end of the cell protrusions. In addition, colocalization between F-actin and PMCA4b was observed at the lamellipodia and cell–cell contact sites that may indicate a direct or indirect interaction between these proteins, as suggested previously [69,70].

Using recovery after photobleaching (FRAP) measurements, no significant change in F-actin level or actin recovery rate was detected in response to PMCA4b expression suggesting that actin polymerization was not affected. Several studies have reported that an increase in intracellular Ca^2+^ concentration can destroy the cortical actin cytoskeleton with changes in cell shape in different cell types [5,44,45,46]. In this study we found that persistent increase in near-actin Ca^2+^ concentration—tested by the genetically encoded Ca^2+^ sensor fused to actin (GCaMP2-actin)—in response to Ca^2+^ ionophore treatment resulted in actin cytoskeleton collapse and cell rounding in the parental cells or in cells expressing the non-functional PMCA4b-DE. In contrast, the functional PMCA4b was able to protect the actin cytoskeleton from Ca^2+^ overload suggesting that PMCA4b can act as a negative modulator of Ca^2+^ induced F-actin depolymerization.

It has been suggested that an increased level of intracellular calcium can trigger cell motility by regulating proteins that interact with the actin cytoskeleton. Cofilin is an actin severing protein that catalyzes actin depolymerization and mediates actin polymerization by the formation of new barbed ends and supplying G-actin monomers. The activity of cofilin is regulated by Ca^2+^, and was found to control lamellipodia and invadopodia formation [19]. A previous study reported the role of CRAC channels in lamellipodia formation through the regulation of cofilin activity by Ca^2+^ [5]. In our study, we observed close localization of GFP-PMCA4b and mCherry-cofilin in A375-GFP-PMCA4b cells at the lamellipodia compared to cofilin localization at the protrusions of the parental and GFP-PMCA4b-DE transfected A375 cells. This may indicate the importance of PMCA4b in localizing cofilin to the leading edge where it may inhibit cofilin activity by reducing nearby Ca^2+^ levels resulting in a less motile cell type. While PMCA4b expression at the leading edge induced cofilin relocalization, several studies indicate that inhibition or knocking down cofilin can reduce cell polarity [20,71]. Interestingly, cofilin can regulate store operated Ca^2+^ entry in platelets through dynamic F-actin remodeling [72]. Further studies are needed to test if cofilin affects PMCA4b localization, and consequently the establishment of the Ca^2+^ gradient in migrating cells.

An interesting point is that PMCA4b expression could affect matrix metalloproteinase (MMP) production because these proteins are calcium dependent zinc-containing endopeptidases, which are essential for the degradation of the extracellular matrix and hence they affect cell migration and metastasis. In correlation with this assumption many previous studies showed the involvement of Ca^2+^ channels in the production of MMPs. For example, an increase in the expression of the Ca^2+^ channels TRPV2 in prostate cancer, and TRPM8 in squamous cell carcinoma resulted in induction of MMP-2 and MMP-9, respectively [73].

It has been reported that localization of PMCA4b at the leading edge was responsible for maintaining the Ca^2+^ gradient and directional movement of HUVEC cells. The PMCA4b mediated high Ca^2+^ pumping rate resulted in a low basal Ca^2+^ level at the cell front that enabled effective local Ca^2+^ signaling by the STIM1/ORAI Ca^2+^ entry channels [48]. In line with these findings, we found that high PMCA4b levels at the cell front also resulted in a typical front-to-rear Ca^2+^ concentration gradient in A375 cells. This change in Ca^2+^ distribution can contribute to the actin-based shape change and PMCA4b induced switch in motility style of BRAF mutant melanoma cells.

Several lines of evidence suggested that non-cancerous epithelial cells express PMCA4b at a relatively high level that is lost or downregulated during tumorigenesis. Using two different cell types, we demonstrate here that the loss of PMCA4b can have a profound effect on cell shape and cell culture morphology through F-actin rearrangement. On the one hand, we show how a highly aggressive melanoma cell type with neural crest origin changes cell shape through actin cytoskeleton remodeling in response to PMCA4b expression. On the other hand, we demonstrate how the ER+ luminal breast cancer epithelial cell-type MCF-7 responds to overexpression or silencing of PMCA4b. Our findings for the role of PMCA4b in actin cytoskeleton remodeling using these two distinct types of cells may give a greater perspective for future studies.

Our data suggest that PMCA4b plays a critical role in regulating cell polarity through F-actin rearrangement that could associate with less aggressive cancer cell phenotype. This is in good agreement with our previous findings that identified PMCA4b as a putative metastasis suppressor in A375 melanoma cells [26]. In general, metastasis suppressors are hardly druggable since they usually disappear from the system during tumor progression. Therefore, our aims have been finding drugs that could counteract with the downregulation of PMCA4b that may help finding ways to prevent metastasis. Besides the conventional drugs targeting BRAF, vemurafenib and dabrafenib, we identified the stress response kinase p38 MAPK as a potential target. We found that PMCA4b is degraded in BRAF mutant cells in a p38 MAPK dependent manner and that specific inhibition of this kinase prevented its degradation [27]. Importantly, p38 inhibitors enhanced PMCA4b expression without affecting cell growth that could make them eligible to fulfill the requirements for the recently proposed group of drugs, “migrastatics” [74]. Another possible option could be the use of the epigenetic drugs, the HDAC inhibitors vorinostat (Zolinza) and/or valproic acid alone or in combination that has been shown to increase PMCA4b expression in a variety of melanoma and breast cancer tumor cells including A375 and MCF-7 [28,29,75].

## 5. Conclusions

Our findings indicate that both the expression and proper trafficking are essential for the antimigratory activity of the PMCA4b pump in BRAF mutant melanoma cells. We suggest that polarized distribution of a fully functional PMCA4b can generate and maintain a front-to-rear increasing Ca^2+^ concentration gradient, and induce redistribution of polymerized F-actin and cofilin from the dynamic protrusions to the leading edge, formation of stable stress fibers, increased cell–cell connections, and decreased vinculin expression resulting in a slow motility melanoma cell type. Manipulating PMCA4b abundance also induced characteristic redistribution of actin filaments in the MCF-7 breast cancer cells suggesting that downregulation of PMCA4b expression during carcinogenesis may contribute to aberrant cancer cell migration and tumor metastasis in different cancer types.

## Data Availability

Data is contained within the article or Supplementary Material.

## Supplementary Materials

The following are available online at https://www.mdpi.com/2072-6694/13/6/1354/s1, Figure S1: Complementary to Figure 1, Figure S2: Complementary to Figure 1D, Figure S3: PMCA4b activity is necessary for the stress fiber formation in A375 melanoma cells, Figure S4: Complementary to Figure 5, Figure S5: Complementary to Figure 6, Figure S6: Complementary to Figure 7, Figure S7: Complementary to Figure 8, Figure S8: Uncropped Western Blot Figures, Videos S1: Complementary to Figure 1D, Videos S2: Complementary to Figure 1D, Video S3: Complementary to Figure 2B, Video S4: Complementary to Figure 4D, Video S5: Complementary to Figure 7B, Video S6 Complementary to Figure 7.
