# Supplementary material for: The Plasma Membrane Ca2+ Pump PMCA4b Regulates Melanoma Cell Migration through Remodeling of the Actin Cytoskeleton
Source: Cancers (Basel). 2021 Mar 17;13(6):1354. doi: 10.3390/cancers13061354 (PMC8002435; doi:10.3390/cancers13061354)

Supplementary Materials: The Plasma Membrane Ca^2+^ Pump PMCA4b Regulates Melanoma Cell Migration Through Remodeling of the Actin Cytoskeleton

Randa Naffa, Rita Padányi, Attila Ignácz, Zoltán Hegyi, Bálint Jezsó, Sarolta Tóth, Karolina Varga, László Homolya, Luca Hegedűs, Katalin Schlett and Agnes Enyedi


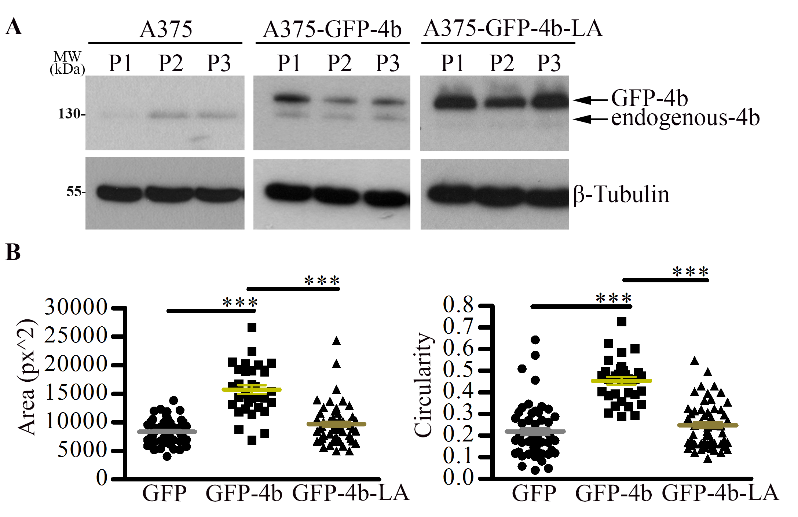


**Figure S1.** Complementary to Figure 1. (**A**) A375, A375-GFP-PMCA4b, A375-GFP-PMCA4b-LA cells were cultured in 6-well plates and total protein was extracted from cells harvested after consecutive three passages (P1, P2, P3). Protein expression of PMCA4b was analyzed by Western bloting. β-tubulin was used as loading control. (**B**) Area and circularity parameters for A375-GFP (n= 56), A375-GFP-PMCA4b (*n* = 32) and A375-GFP-PMCA4b-LA cells (*n* = 54) were analyzed by the ImageJ software.


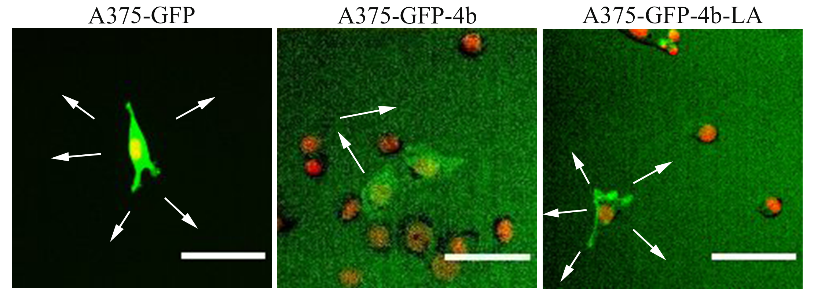


**Figure S2.** Complementary to Figure 1D. Single cell images of A375-GFP, A375-GFP-PMCA4b and A375-GFP-PMCA4b-LA cells were taken by acquiring GFP and Hoechst signals in every 30 minutes for 24 hours. Videos were created from the sequential images (Video S2). Scale bar, 50 µm.


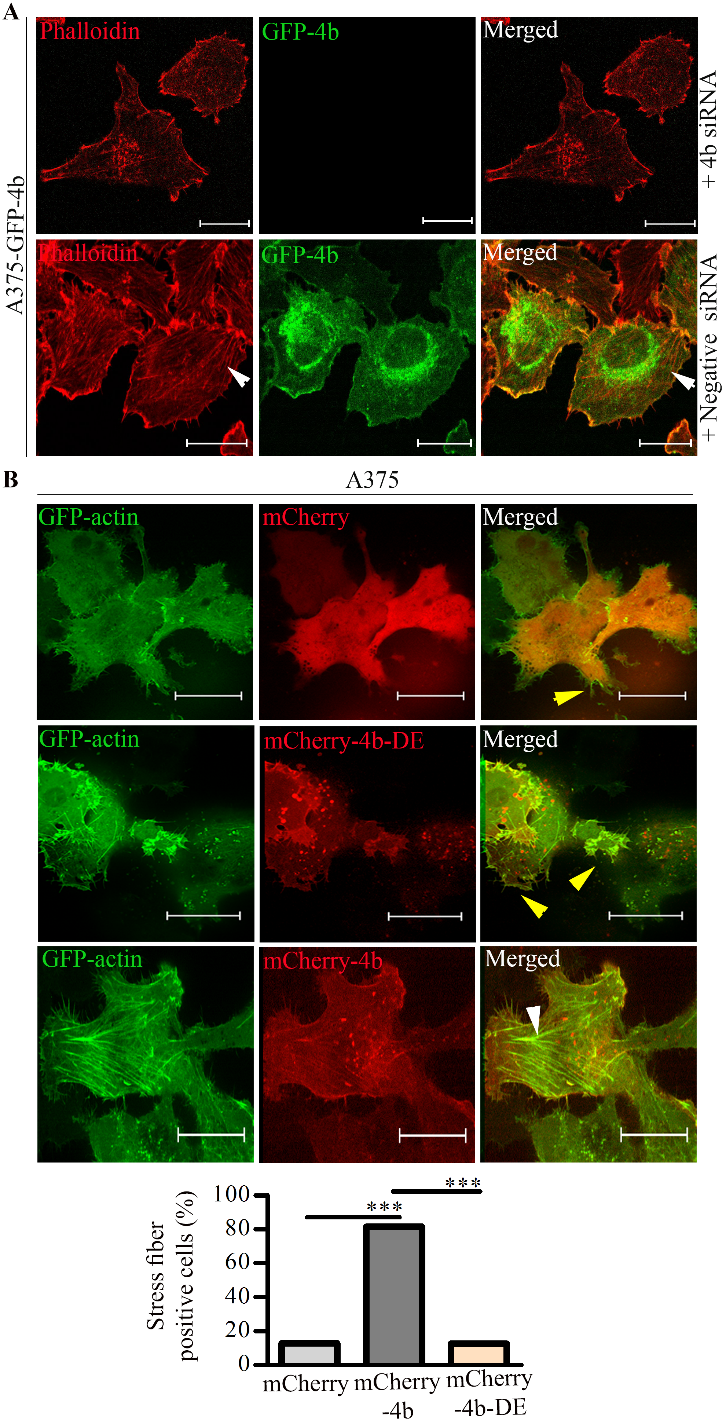


**Figure S3.** PMCA4b activity is necessary for the stress fiber formation in A375 melanoma cells. (**A**) complementary to Figure 3A. A375-GFP-PMCA4b cells were transfected with ON-Target plus SMARTpool PMCA4b (ATP2B4) siRNA or SignalSilence^®^ control siRNA (50 nM, Cell Signalling Technology, cat. #65685) as indicated and incubated for 72 hours. Cells were labelled with Phalloidin –TRITC. Arrowheads show stress fibers. Scale bar, 20 µm. (**B**) A375 cells were transfected with GFP-actin together with one of the following plasmids, pmCherry-C1, mCherry-PMCA4b or mCherry-PMCA4b-DE, and cultured for 48 hours. Cells were fixed and confocal microscopy images were taken for the bottom of the cells. Yellow arrowheads show actin spikes and white arrowheads show stress fibers. Scale bar, 20 µm. The fractions of stress fiber-positive cells for GFP-actin and mCherry (*n* = 31), mCherry-PMCA4b (*n* = 43) and mCherry-PMCA4b-DE (*n* = 48) were determined.


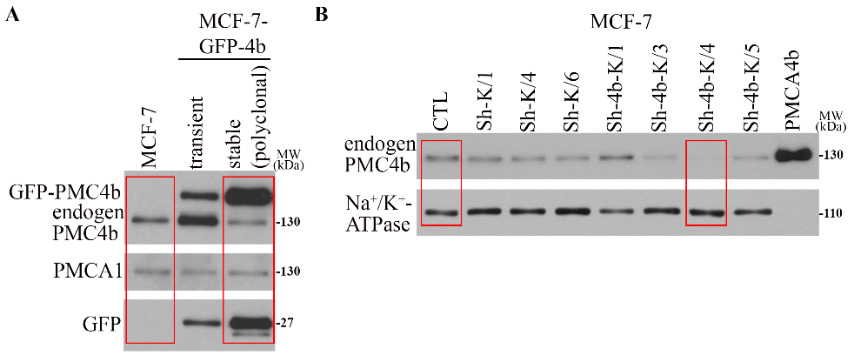


**Figure S4.** Complementary to Figure 5. (**A**,**B**) MCF7, MCF7-GFP-PMCA4b and MCF7-Sh-PMCA4b cells were cultured in a 6-well plate for 48 hours. Protein expression from total cell lysate was analyzed by Western blotting. PMCA1 or NA^+^/K^+^ ATPase were used as loading controls. Red boxes indicate the cells used in the experiment shown in Figure 5.


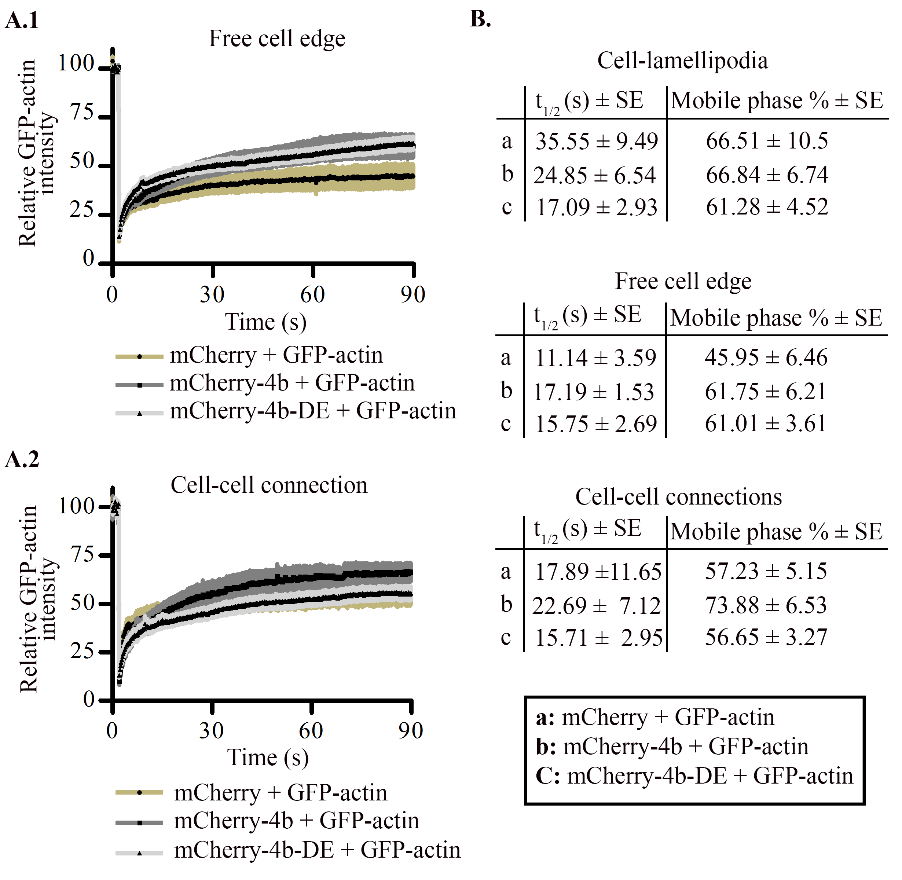


**Figure S5.** Complementary to Figure 6. (A.1+A.2) This is the same experiment as described for figure 6. However, the GFP-actin was photobleached at the cell free edge (n=9, n=12, n=15) and at the cell connections (*n* = 5, *n* = 8, *n* = 12) for cells expressing either mCherry, mCherry-4b or mCherry-4b-DE, respectively, using spinning-disc confocal microscopy. GFP signal was recorded every 0.2 sec for 90 seconds at 37 ºC. Relative GFP-actin fluorescence intensity was calculated and data was presented using GraphPad Prism software v5.01. Data represent mean ± S.E.M for three independent experiments. (**B**) T_1/2_ and mobile phase were calculated for all the ROIs taken for the cell ruffles (Figure 6), cell edge free (A.2) and cell connections (B.2) using GraphPad Prism software v5.01.


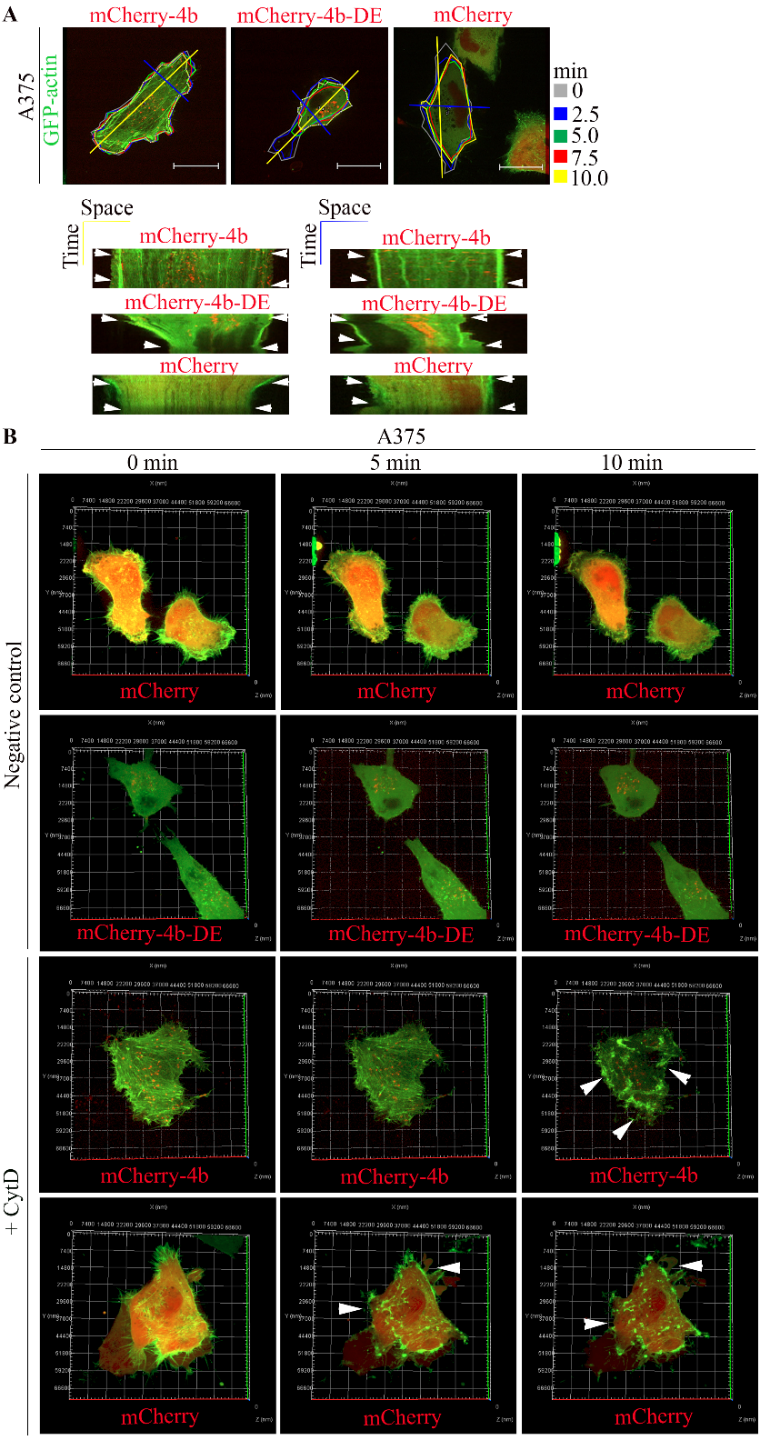


**Figure S6.** Complementary to Figure 7. (**A**) This is the same experiment as described for Figure 7A. Lines were drawn along the cells and a kymograph was generated using ImageJ software. Arrowheads indicate changes in cell edge position of the same space with time. (**B**) For positive control, A375 cells transiently expressing pmCherry-C1 or mCherry-PMCA4b together with GFP-actin were treated with 2.5 µM cytD. For negative control, A375 cells transiently expressing pmCherry-C1 or mCherry-DE were treated with 2 µM A23187 in a HBSS buffer without Ca^2+^ containing 100 µM EGTA. The cells were treated for 10 minutes at 37 ºC. A 3D confocal microscopy images were taken at 0, 5 and 10 minutes after A23187 addition using spinning-disc confocal microscopy. Arrowheads indicate changes in cell shape.


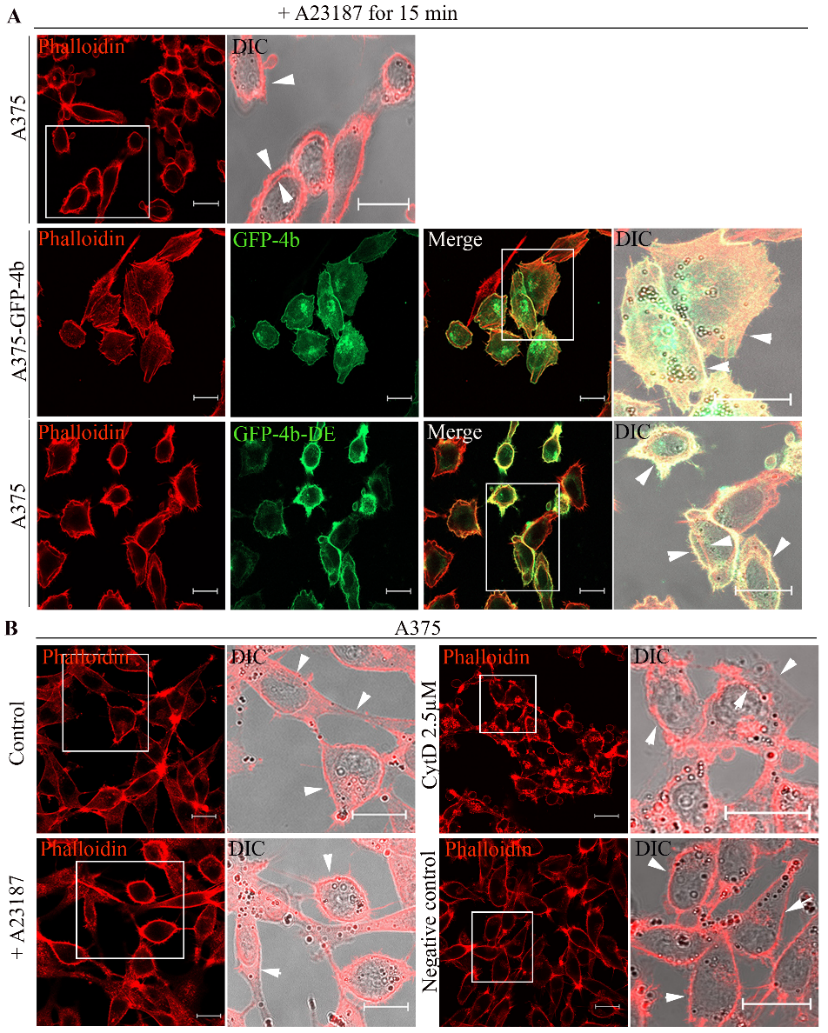


**Figure S7.** Complementary to Figure 8. (**A**) The same experiment in Figure 8, cells were treated with 2 µM of A23187 for 15 min at 37 ºC. scale bar, 20 µm. (**B**) A375 cells were cultured for 48 hours then cells were treated with 2 µM A23187 in HBSS buffer with or without (negative control) Ca^2+^ or 2.5 µM cytD, as indicated. Scale bar, 20 µm. (**A,B**) Confocal microscopy images of cells labelled with Phalloidin-TRITC. Insets show cells with higher magnification. Arrowheads show the position of actin in relation to the cell periphery. Scale bar, 20 µm.

**Figure S8.** Uncropped western Blots Figures

Figure 3C Western blot


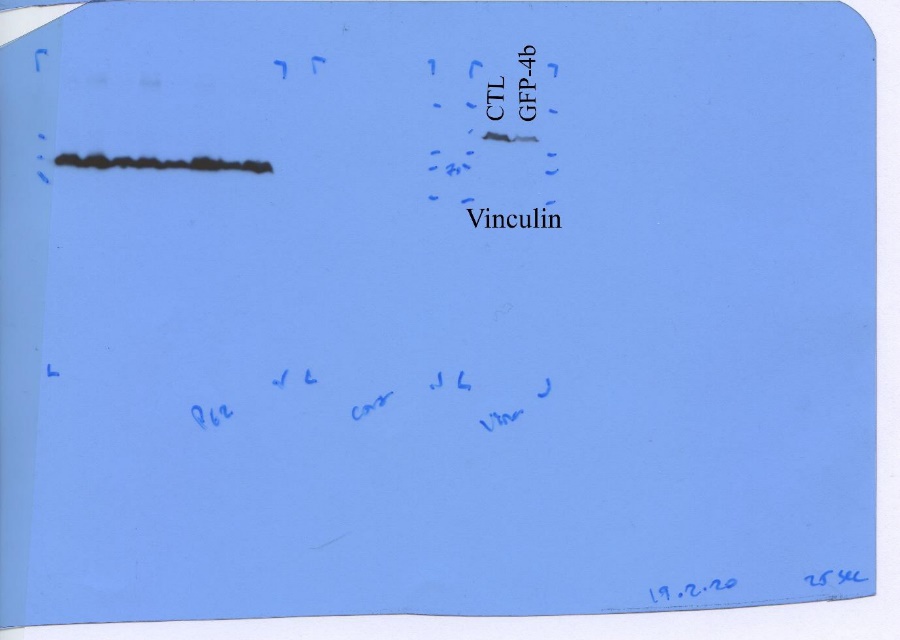


Figure 3C Western blot.


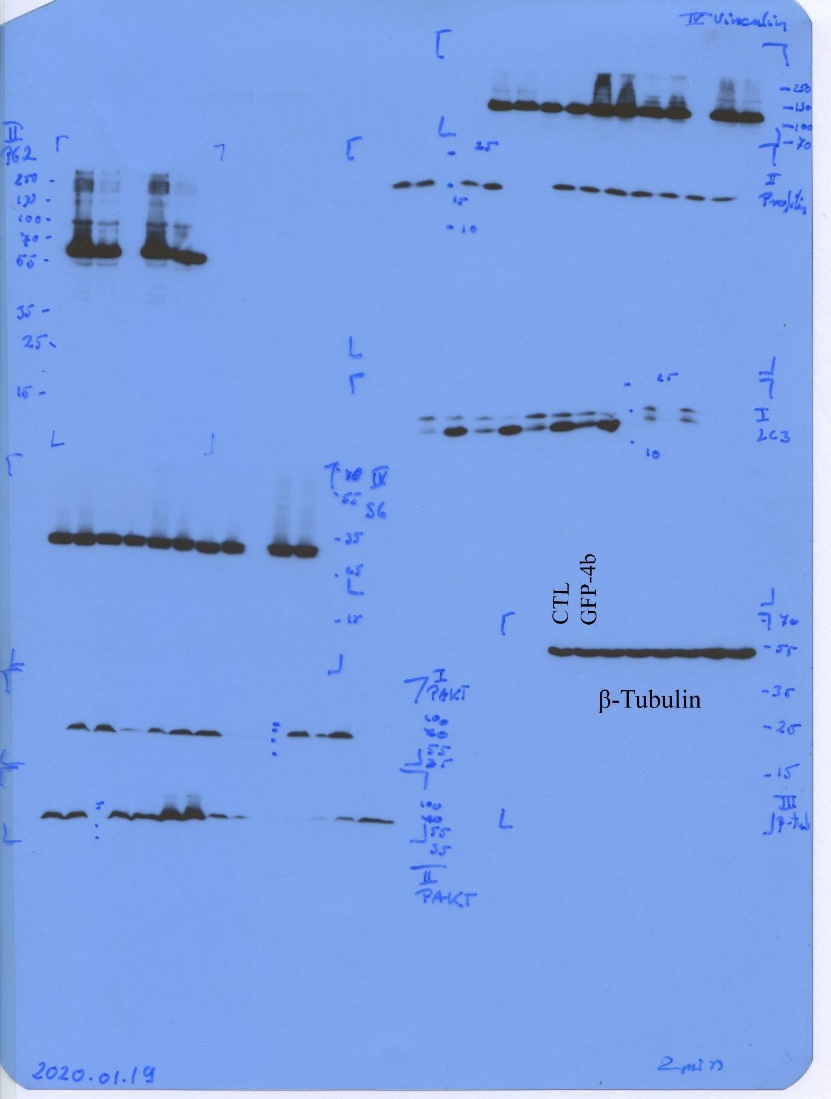


Figure 9B Western blot

.
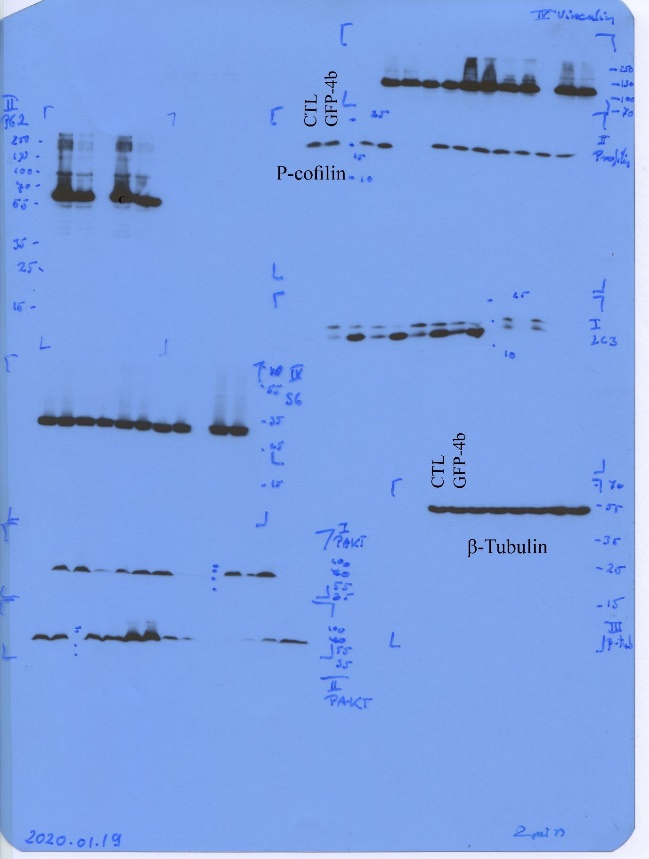


Figure S1A Western blot.


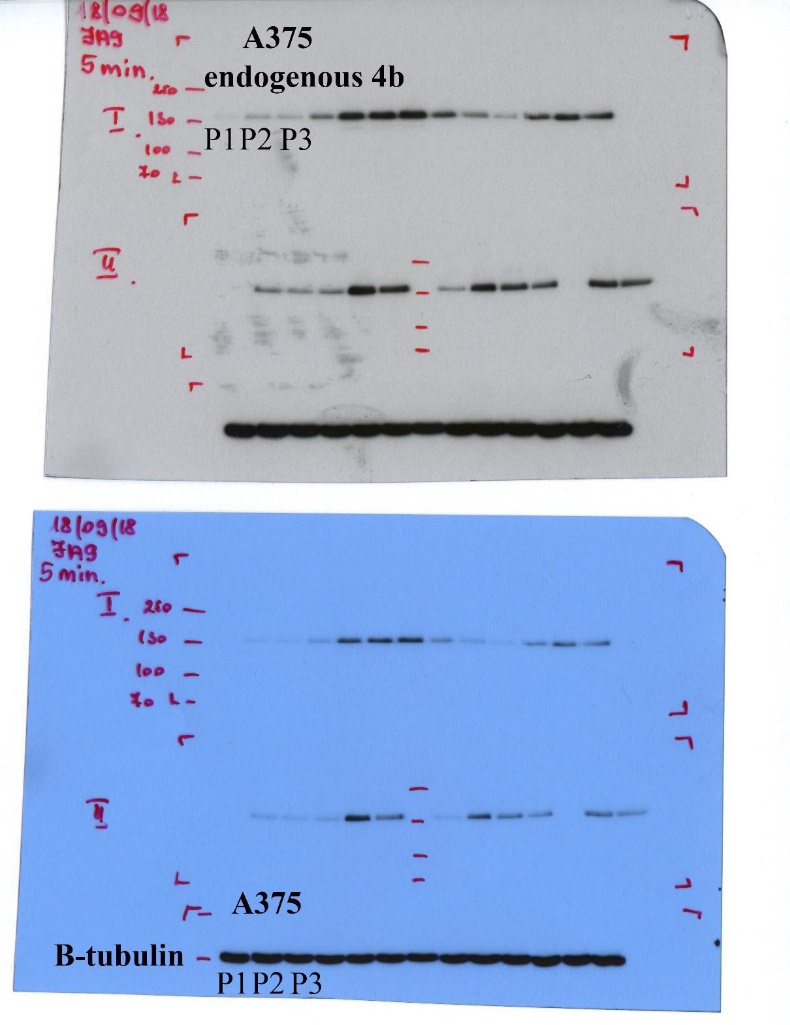


Figure S1A Western blot.


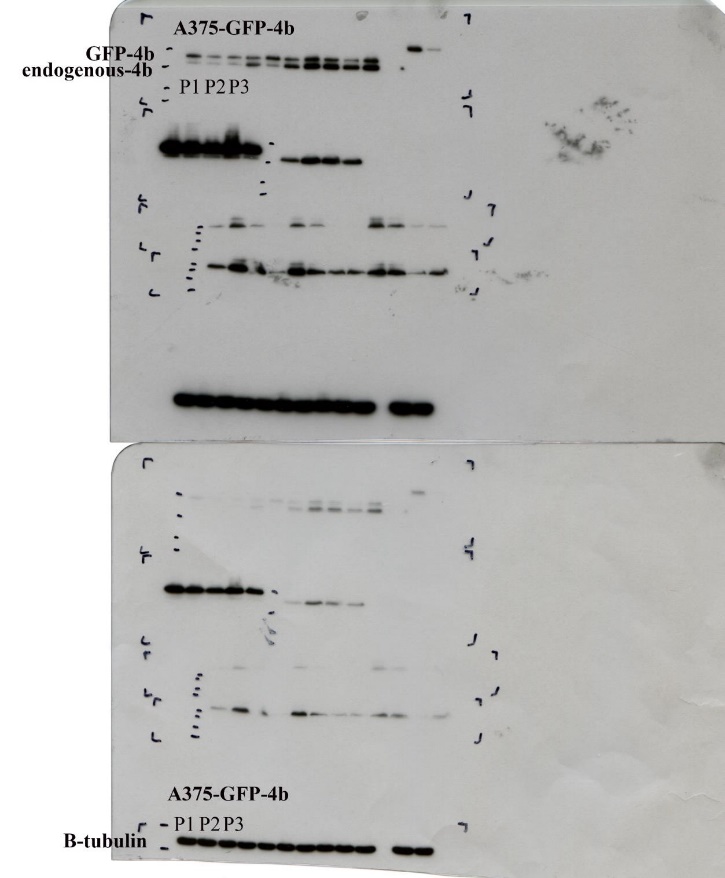


Figure S1A Western blot.


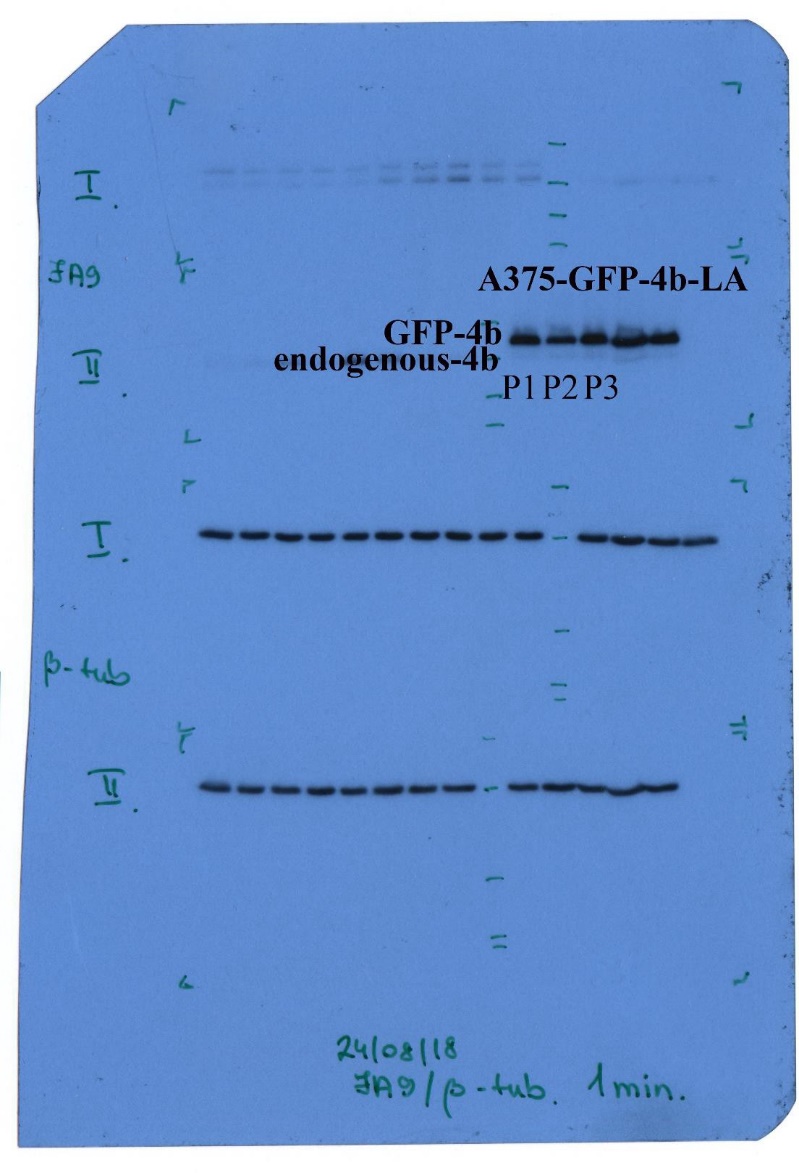


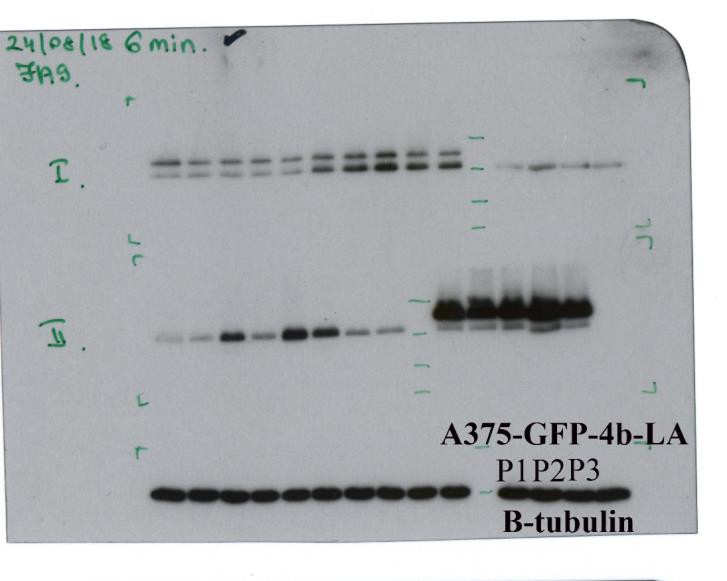


Figure S4A Western blot


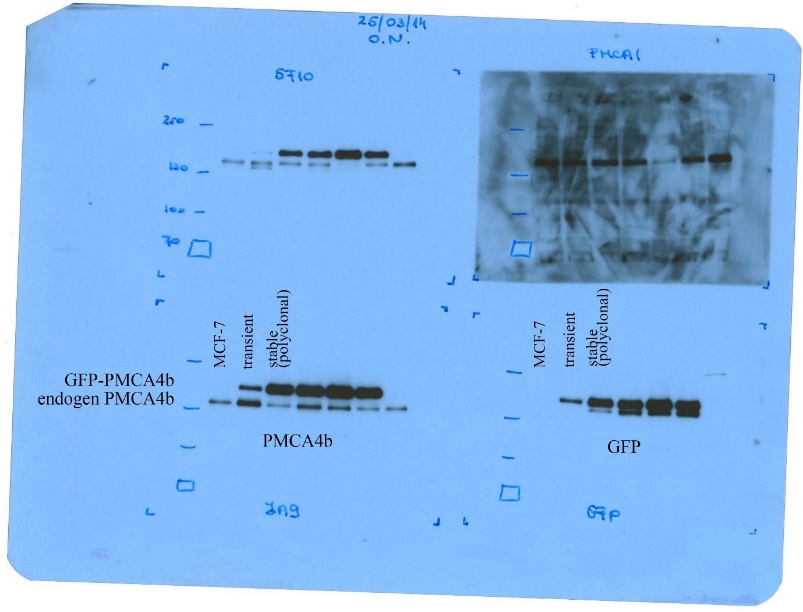


Figure S4A Western blot


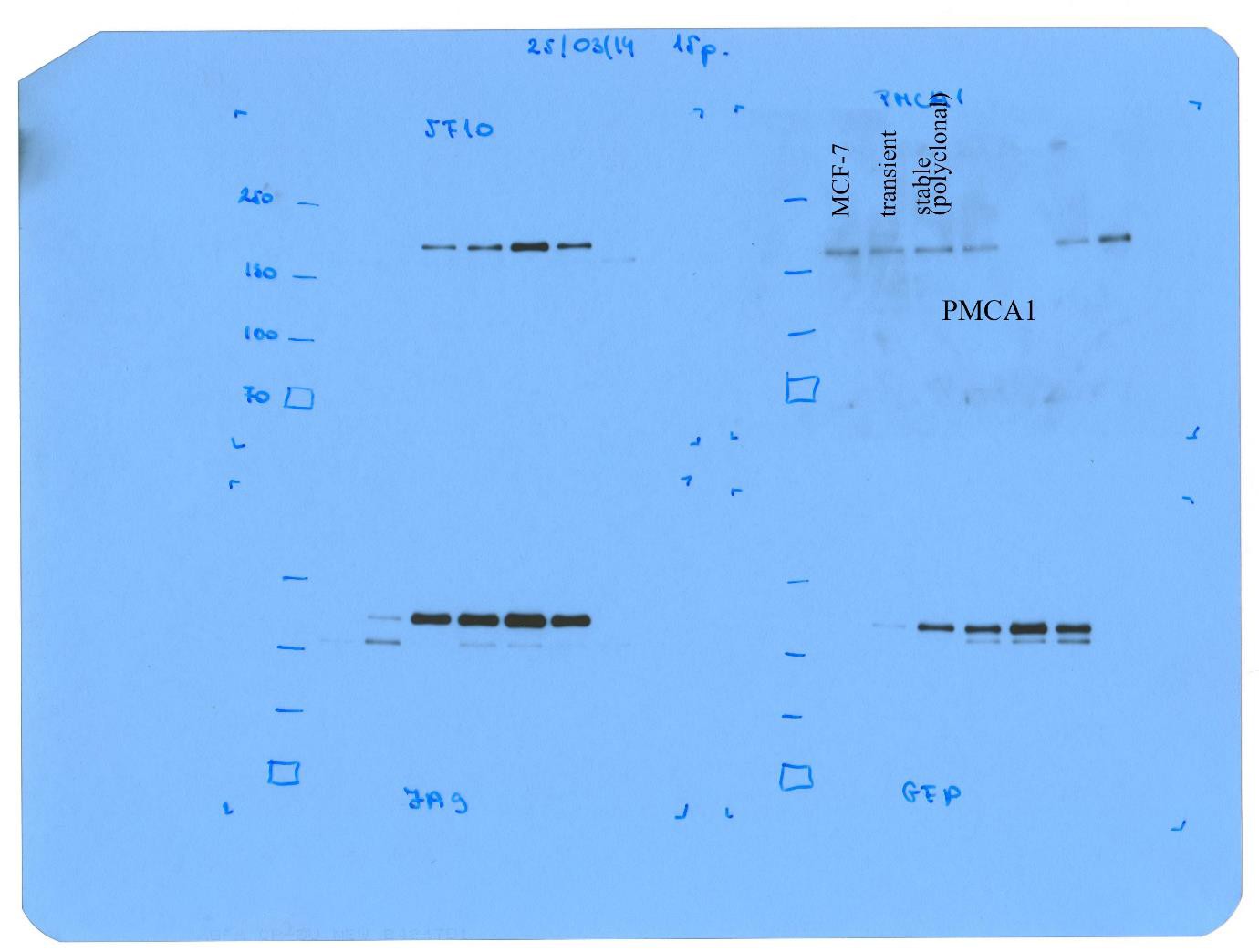


Figure S4B Western blot


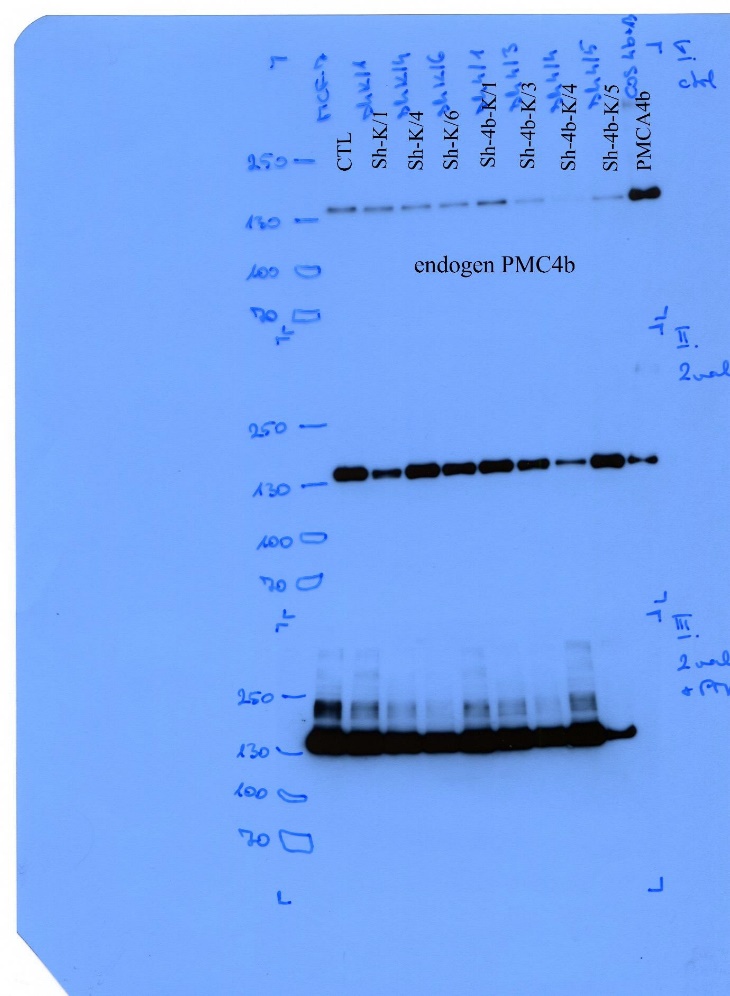


Figure S4B Western blot


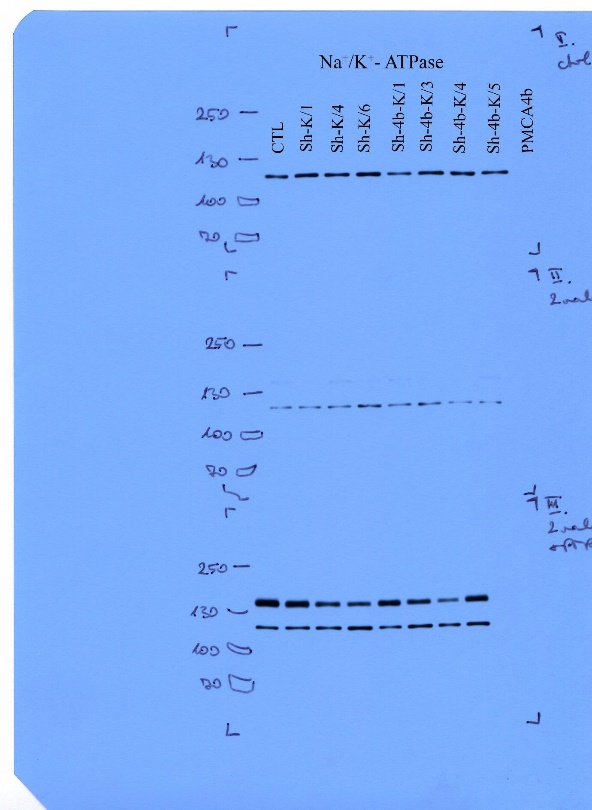

Supplement: Supplementary file 1 [file cancers-13-01354-s001.zip › cancers-1110287-supplementary figures.docx]
